# Evaluating the fate of bacterial indicators, viral indicators, and viruses in water resource recovery facilities

**DOI:** 10.1002/wer.1096

**Published:** 2019-04-20

**Authors:** Thomas Worley‐Morse, Melanie Mann, Wendell Khunjar, Lola Olabode, Raul Gonzalez

**Affiliations:** ^1^ Hazen and Sawyer Lakewood Colorado; ^2^ Hazen and Sawyer Raleigh North Carolina; ^3^ Hazen and Sawyer Fairfax Virginia; ^4^ Water Research Foundation Alexandria Virginia; ^5^ Hampton Roads Sanitation District Virginia Beach Virginia

**Keywords:** coliphage, indicator, recreational water quality criteria, virus, wastewater

## Abstract

A year‐long sampling campaign at nine water resource recovery facilities (WRRFs) was conducted to assess the treatability and fate of bacterial indicators, viral indicators, and viruses. Influent concentrations of viral indicators (male‐specific and somatic coliphages) and bacterial indicators (*Escherichia coli* and enterococci) remained relatively constant, typically varying by one order of magnitude over the course of the year. Annual average bacterial indicator reduction ranged from 4.0 to 6.7 logs, and annual average viral indicator reduction ranged from 1.6 to 5.4 logs. Bacterial and viral indicator reduction depended on the WRRF's treatment processes, and bacterial indicator reduction was greater than viral indicator reduction for many processes. Viral reduction (adenovirus 41, norovirus GI, and norovirus GII) was more similar to viral indicator reduction than bacterial indicator reduction. Overall, this work suggests that viral indicator reduction in WRRFs is variable and depends on specific unit processes. Moreover, for the same unit treatment process, viral indicator reduction and bacterial indicator reduction can vary.

**Practitioner points:**

- A year‐long sampling campaign was conducted at nine water resource recovery facilities (WRRFs).
- The treatability and fate of bacterial indicators, viral indicators, and viruses were assessed.
- Viral indicator reduction in WRRFs is variable and depends on specific unit processes.
- For the same unit treatment process, viral indicator reduction and bacterial indicator reduction can vary.

## Graphical abstract

A year‐long sampling campaign at nine water resource recovery facilities (WRRFs) was conducted to assess the treatability and fate of bacterial indicators (*Escherichia coli* and enterococci), viral indicators (male‐specific and somatic coliphages), and viruses (adenovirus 41, norovirus GI, and norovirus GII). Bacterial and viral indicator reduction depended on the WRRF's treatment processes, and bacterial indicator reduction was greater than viral indicator reduction for many processes. Viral reduction was more similar to viral indicator reduction than bacterial indicator reduction. This work suggests that viral indicator reduction in WRRFs is variable and depends on specific unit processes.

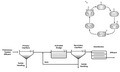

## Introduction

bacteriophages are viruses that infect bacteria, and coliphages are a class of bacteriophages that infect *Escherichia coli*. The desire to use coliphages as viral indicators for fecal water contamination is motivated by research findings that suggest viral pathogens are significant causative agents of gastroenteric disease in recreational waters and that coliphages better mimic the fate of enteric viruses than indicator bacteria (Costán‐Longares et al., 2008; Jiang, Chu, & He, 2007; Jofre, Lucena, Blanch, & Muniesa, 2016; Lee, Dawson, Ward, Surman, & Neal, 1997; Sinclair, Jones, & Gerba, 2009; Soller, Bartrand, Ashbolt, Ravenscroft, & Wade, 2010). For instance, recent work by the Centers for Disease Control (CDC) suggested that viral exposure was linked to approximately 36% and 44% of the untreated recreational water acquired illnesses reported to the CDC from 2009 to 2010 and from 2011 to 2012, respectively (Hlavsa et al., 2014, 2015). Others have estimated higher values of up to 56% based on quantitative microbial risk assessments (Soller et al., 2010).

The U.S. Environmental Protection Agency (USEPA) is evaluating coliphages as indicators for future updates of USEPA's Recreational Water Quality Criteria (RWQC; USEPA, 2015). Changes to RWQC can directly impact water resource recovery facilities (WRRFs) that discharge into primary contact recreational waters. For example, coliphage RWQC could result in state water quality standards for coliphage, which in turn would require state permit writers to develop National Pollution Discharge & Elimination System (NPDES) coliphage effluent limits for WRRFs, which may require treatment changes to meet low‐level effluent coliphage concentrations. The magnitude of the impact on the wastewater industry is unclear because limited data exist on the fate and treatability of the indigenous coliphages present in wastewater (Amarasiri, Kitajima, Nguyen, Okabe, & Sano, 2017; Pouillot et al., 2015; Rose et al., 2004). For example, Pouillot et al. (2015) performed a meta‐analysis of male‐specific coliphages in WRRFs and found large variations in coliphage reduction between different WRRFs; however, the study focused on whole plant removal for a few process configurations and disinfectants. Amarasiri et al. (2017) reviewed previous studies on coliphage removal in various processes, which included membrane bioreactors (MBR), activate sludge, constructed wetlands, pond systems, microfiltration, and ultrafiltration, and concluded that coliphages serve as suitable viral indicators and that additional data are needed to confirm the indicator reduction for each specific wastewater process. Rose et al. (2004) reviewed the fate of indicators and pathogens at multiple WRRFs and concluded that wide discrepancies exist on indicator and pathogen removal between WRRFs and that additional information was needed on indicator seasonality trends. Additionally, others have suggested that bacteriophages perform satisfactorily as indicators of fecal contamination and virus removal in wastewater treatment (García‐Aljaro, Blanch, Campos, Jofre, & Lucena, 2018; McMinn, Ashbolt, & Korajkic, 2017).

Given these uncertainties, our goal was to evaluate the fate and persistence of bacterial indicators (*E. coli* and enterococci) viral indicators (male‐specific and somatic coliphages), and enteric viruses (adenovirus 41, norovirus GI, and norovirus GII) at WRRFs using different treatment configurations and disinfectants. To achieve this goal, a year‐long sampling campaign was conducted at nine WRRFs located throughout the United States to (a) determine how the influent concentrations of indicators vary over the course of a year; (b) determine the fate and persistence of viral and bacterial indicators at WRRFs and to determine the process parameters that affect indicator removal; (c) evaluate the fate of enteric viruses versus bacterial and viral indicators in WRRFs (at three of the nine WRRFs); and (d) determine which types of WRRFs would likely be most affected by coliphage RWQC.

## Methods

### Water resource recovery facilities

A year‐long sampling campaign was conducted at nine WRRFs located in the following US regions: west, south, and northeast. Although the participating WRRFs do not all identify as WRRFs, each is referred to as a WRRF in this study. For each WRRF, *E. coli*, enterococci, male‐specific coliphages, and somatic coliphages were enumerated at three to five locations in the treatment process each month over the course of 1 year. WRRF process information is provided in Table 1, and sampling locations are provided in Supporting Information Table S1. The raw influent sampling locations were located upstream of facility recycle streams, such as filter backwash water and dewatering processes effluents, which may be recycled to the head of the facility.

**Table 1:** WRRF process configuration and indicator reduction

WRRF name	Process configuration	Indicator organism	Mean Influent Conc. (Log Units/100 ml)	Mean Effluent Conc. (Log Units/100 ml)	Mean log reduction
WRRF A	5‐Stage BNR with LPHO UV	*E. coli*	6.27 ± 0.16	0.88 ± 0.610	5.39 ± 0.57
		Enterococci	5.43 ± 0.34	0.62 ± 0.57	4.81 ± 0.54
		Somatic	5.29 ± 0.55	0.21 ± 0.36	5.08 ± 0.64
		Male‐specific	4.81 ± 0.23	0.13 ± 0.20	4.68 ± 0.30
WRRF B	5‐Stage BNR with tertiary clarification (ferric), dual train filtration (deep bed and UF GAC/BAC), and ozone	*E. coli*	6.51 ± 0.19	0.18 ± 0.64	6.32 ± 0.65
		Enterococci	6.02 ± 0.09	0.11 ± 0.39	5.91 ± 0.46
		Somatic	5.21 ± 0.18	0.00 ± 0.00	5.21 ± 0.18
		Male‐specific	5.19 ± 0.13	0.00 ± 0.00	5.19 ± 0.13
WRRF C	5‐Stage BNR with MBR and MP UV	*E. coli*	6.75 ± 0.30	0.08 ± 0.28	6.67 ± 0.43
		Enterococci	6.20 ± 0.13	0.08 ± 0.28	6.12 ± 0.33
		Somatic	5.36 ± 0.23	0.00 ± 0.00	5.36 ± 0.23
		Male‐specific	4.93 ± 0.27	0.00 ± 0.00	4.93 ± 0.27
WRRF D	Aerated grit (no primary clarifiers) with step‐aeration activated sludge and sodium hypochlorite	*E. coli*	6.76 ± 0.24	1.87 ± 0.36	4.89 ± 0.39
		Enterococci	6.19 ± 0.22	0.63 ± 0.60	5.56 ± 0.61
		Somatic	5.26 ± 0.23	2.69 ± 0.55	2.57 ± 0.68
		Male‐specific	5.22 ± 0.79	3.18 ± 0.62	2.04 ± 0.91
WRRF E	Pure oxygen activated sludge with sodium hypochlorite	*E. coli*	6.84 ± 0.34	1.44 ± 0.53	5.40 ± 0.63
		Enterococci	6.24 ± 0.27	0.23 ± 0.42	6.01 ± 0.65
		Somatic	5.46 ± 0.39	3.45 ± 0.45	2.01 ± 0.43
		Male‐specific	4.96 ± 0.26	3.27 ± 0.37	1.69 ± 0.38
WRRF F	Sequencing batch reactor with peracetic acid	*E. coli*	6.32 ± 0.45	1.73 ± 0.57	4.59 ± 0.68
		Enterococci	5.84 ± 0.29	1.93 ± 1.02	3.91 ± 1.18
		Somatic	5.55 ± 0.57	2.74 ± 0.59	2.81 ± 0.84
		Male‐specific	5.06 ± 0.41	2.85 ± 0.50	2.21 ± 0.49
WRRF G	Integrated fixed‐film activated sludge with sodium hypochlorite	*E. coli*	6.47 ± 0.11	0.36 ± 0.26	6.11 ± 0.28
		Enterococci	5.59 ± 0.10	0.04 ± 0.14	5.55 ± 0.18
		Somatic	4.88 ± 0.43	1.61 ± 0.68	3.27 ± 0.66
		Male‐specific	5.12 ± 0.21	1.50 ± 0.85	3.61 ± 0.90
WRRF H	Three‐cell activated sludge with swing/anoxic aerobic for the first cell with sodium hypochlorite	*E. coli*	6.22 ± 0.74	0.61 ± 0.59	5.60 ± 0.97
		Enterococci	5.58 ± 0.42	0.24 ± 0.44	5.34 ± 0.47
		Somatic	4.77 ± 0.43	1.40 ± 0.47	3.38 ± 0.67
		Male‐specific	4.84 ± 0.14	1.23 ± 0.51	3.61 ± 0.54
WRRF I	3‐Stage BNR (A2O) with sodium hypochlorite	*E. coli*	6.41 ± 0.63	0.45 ± 0.50	5.96 ± 0.82
		Enterococci	5.50 ± 0.26	0.29 ± 0.36	5.21 ± 0.51
		Somatic	4.59 ± 0.35	0.59 ± 0.77	4.00 ± 0.80
		Male‐specific	4.64 ± 0.31	0.63 ± 0.93	4.01 ± 0.98

Secondary processes included both biological nutrient removal (BNR) processes and non‐BNR processes. The two non‐BNR processes were pure oxygen activated sludge and high‐rate activated sludge with step feed. BNR processes included integrated fixed‐film activated sludge (IFAS), three‐cell activated sludge with a swing anoxic/aerobic cell, 3‐stage anaerobic–anoxic–aerobic (A2O), 5‐stage BNR, sequencing batch reactor (SBR), and an MBR. The selected WRRFs represented the major wastewater disinfectants: chlorine, ultraviolet (UV) light, ozone, and peracetic acid (PAA). If the WRRF uses tertiary treatment, the WRRF sampled after tertiary treatment in lieu of sampling after primary treatment. WRRFs B and C have tertiary processes, so primary effluent samples were not collected, and WRRFs D and E do not have primary treatment.

### Sample and data collection

At each sample location for WRRFs A through F, a one liter grab sample was aseptically collected for analysis. Sodium thiosulfate was used for quenching of disinfectant residuals at a final concentration of 100 mg/L. For Facilities A through F, samples were collected mid‐morning, stored on ice, and shipped overnight to the contract laboratory for analysis (Scientific Methods Inc., IN).

WRRFs G, H, and I, which used their utility's laboratory, started microbial tests on the same day samples were collected. Four liters of sample was collected at each site in sterile containers, and then partitioned upon arrival at the laboratory for fecal indicator bacteria and coliphage analyses, and for enteric virus concentration and molecular quantification.

Facilities reported wastewater characteristics and operational parameters during sampling events that included influent and effluent flow, influent temperature, influent pH, mixed liquor suspended solids (MLSS), disinfectant dose, and disinfectant residual. The solids residence time (SRT) was calculated for each WRRF. All measurements were performed using Standard Methods for the Examination of Water and Wastewater (APHA, 2017). The participating WRRFs collected most samples during dry weather under normal flows, and samples collected during wet weather events were noted in the respective Supporting Information Tables S1–S24.

### Bacterial and coliphage quantification

*E*. were enumerated by USEPA Method 1603 on modified mTEC agar plates (USEPA, 2014). Enterococci were enumerated by USEPA Method 1600 on mEI agar plates (USEPA, 2006). Coliphages were quantified by the USEPA Method 1602 single agar layer procedure (USEPA, 2001). Serial dilutions were used to obtain the proper concentration for coliphage plating. All bacterial and coliphage data were reported in colony‐forming units (CFU) or plaque‐forming units (PFU) per 100 ml with limits of detection of 1 CFU or PFU per 100 ml. For quality assurance, duplicate sample grabs and analyses were performed on 10% of the samples. Positive and negative controls were performed for the above microbial tests according to the respective USEPA methods. WRRF indicator concentration data are reported in Supporting Information Tables S3 through S20.

### Enteric virus concentration, extraction, and quantification

In addition to the above culture‐based methods, droplet digital PCR (ddPCR) was used to enumerate adenovirus 41, norovirus GI, and norovirus GII gene copies from WRRFs G, H, and I. The primer and probe information used in this study are presented in Supporting Information Table S2.

Mixed cellulose ester HA filters (HAWP04700; Millipore, Billerica, MA, USA) were used to concentrate viral enteric pathogens in 50–1,000 ml water samples. Prior to sample filtration, MgCl_2_ was added to a final concentration of 25 mM, and then the samples were acidified to a pH of 3.5 with 20% HCl. Immediately after filtration, filters were stored in a ‐80°C freezer until total nucleic extraction using NucliSENS easyMag (bioMerieux, Inc., Durham, NC, USA) was completed. Filters were extracted within 14 days of sampling and usually within the same week. Prior to extraction, 10 μl of 1 × 10^6^ copies/μl Hep G Armored RNA (Asuragen, Austin, TX, USA) and 10 μl of 10 μg/ml Salmon Sperm DNA (Sigma‐Aldrich, St. Louis, MO, USA) were spiked in the lysis buffer with all sample and control electronegative filters to quantify matrix inhibition. All extractions were performed according to the manufacturer's protocol B 2.0.1 with modifications. The protocol was modified with a 30‐min off board lysis using 2 ml of lysis buffer and 100 μl of magnetic silica beads to maximize inhibition removal. Using the modified protocol, the samples, standards, and negative extraction control (NEC) were extracted and eluted to a final volume of 100 μl. Human adenovirus 41 (ATCC number VR‐930), norovirus GI (ATCC number VR‐3234SD), and norovirus GII (ATTC number VR‐3235SD) positive genomic RNA or DNA standards were from American Type Culture Collection (ATCC, Manassas, VA, USA).

Molecular ddPCR assays (including the Hepatitis G Armored RNA and Salmon Sperm DNA assays, see Supporting Information Table S2) were analyzed on a Bio‐Rad QX200 (Bio‐Rad, Hercules, CA, USA). For DNA ddPCR, a 20 μl final reaction volume was analyzed. This total volume included 10 μl 2 × ddPCR Supermix for Probes (No dUTP; Bio‐Rad), 3 μl of forward and reverse primers and probes (final concentrations of primers and probes were 900 and 250 nM, respectively), 4 μl of RNase‐free water, and 3 μl of DNA. The reaction mixture was then combined with 70 μl of droplet generation oil in the droplet generator (Bio‐Rad), and the droplets were transferred to a 96‐well plate for PCR amplification using the following conditions: 10‐min enzyme activation at 95°C (1 cycle), 30‐s denaturation at 94°C (40 cycles), 1‐min annealing/extension cycle at 50–55°C (temperature variable depending on optimization; 40 cycles; ramp rate setting to ~2–3°C/s), 10‐min enzyme deactivation at 98°C (1 cycle), followed by an optional hold at 4°C until droplet reading on a droplet reader (Bio‐Rad).

For RNA (one‐step) ddPCR, a 20 μl final reaction volume was comprised of 5 μl 1 × one‐step RT‐ddPCR Supermix (Bio‐Rad), 2 μl of reverse transcriptase (Bio‐Rad), 1 μl of 300 mM DTT, 3 μl forward and reverse primers and probes (final concentrations of primers and probes were 900 and 250 nM, respectively), 5 μl RNase‐free water, and 4 μl of RNA. RNA was denatured at 95°C for 5 min and kept on ice prior to addition to the reaction. The reaction mixture was then mixed with 70 μl droplet generation oil in the droplet generator, and the droplets were transferred to a 96‐well plate for PCR amplification using the following conditions: 60‐min reverse transcription at 50°C (1 cycle), 10‐min enzyme activation at 95°C (1 cycle), 30‐s denaturation at 94°C (40 cycles), 1‐min annealing/extension cycle at 55°C (40 cycles; ramp rate setting to ~2–3°C/s), 10‐min enzyme deactivation at 98°C (1 cycle), followed by an optional hold at 4°C until droplet reading on a droplet reader.

All molecular analysis samples were run in triplicate, and reactions were considered positive if at least three droplets (out of 10,000–20,000) were identified as positive. The threshold was manually set at the lower 1/3 of the space between the negative and positive droplets. Inhibition was determined by calculating the Hepatitis G Armored RNA and Salmon Sperm DNA log reductions in the samples compared to the NEC. For samples where inhibition was an issue, the samples were diluted until there was less than a 0.5 log difference. Using the Hepatitis G RNA and Salmon Sperm DNA, 31.3% (45/144) and 11.1% (16/144) of samples were inhibited prior to dilution, respectively.

### Statistics

Statistical analyses and graphics were performed and generated with OriginPro 2018 (OriginLab Corporation, Northampton, MA, USA) and R (R Core Team, 2017). When culture‐based indicators or molecular enteric virus concentrations were below detection limits, such as for samples from the final disinfected effluent, the value was reported as the detection limit. In the limited cases where the culturable samples were too numerous to count, the upper limit of quantification was used for the concentration and the resulting statistical analyses. In both cases, the data in the supporting information were noted if either the detection limit or the upper limit of quantification was used. Detection limits for molecular assays are presented in Supporting Information Table S2.

Mean values in this report are reported as geometric means. Box‐and‐whisker plots of *E. coli*, enterococci, male‐specific coliphage, and somatic coliphage concentrations (CFU or PFU/100 ml) were constructed for each WRRF, and indicator concentrations were grouped by sampling location. All log calculations are in base 10. Log reductions were calculated by subtracting the log final concentration from the log initial concentration for the process of interest. Spearman's rank‐order correlations were used to determine the degree of association between indicators and pathogens using pooled data from WRRFs G, H, and I at the raw influent and final effluent treatment steps.

To evaluate which process parameters had the strongest relationship with indicator reduction for primary treatment (WRRFs G through I), secondary treatment (all WRRFs), and disinfection processes (WRRFs D through I), linear models were constructed relating indicator reduction to the process variable for the pooled data from each WRRF. Additional process data were collected from WRRFs G through I, which included wastewater nitrogen speciation, phosphorus speciation, salinity, and the chemical oxygen demand. Secondary process removal was related to SRT and the MLSS for all WRRFs. For the disinfection linear models, correlations were developed with the calculated CT values and a forced intercept model was used to ensure that a CT of zero corresponded to no inactivation. No disinfectant correlations were performed for the WRRFs in this study with UV or ozone disinfection due to the low pre‐disinfection influent and final effluent concentrations of all indicators at these WRRFs.

## Results and Discussion

### Influent indicator variability

**Figure 1:**
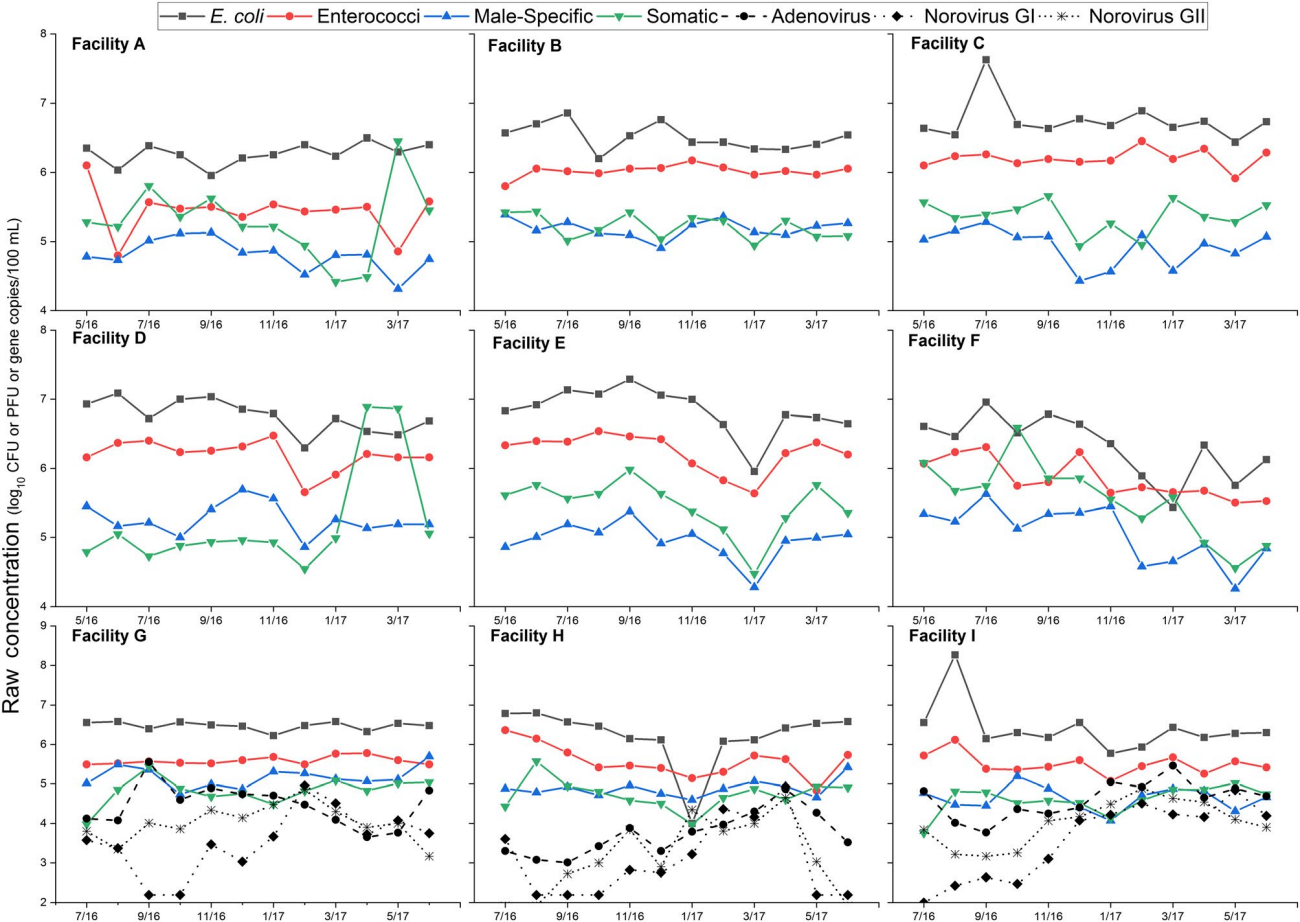
Raw influent concentration of bacterial indicators, viral indicators, and viruses for WRRFs A through I (WRRFs G through I are plotted on different axes).

As noted by others, additional data are needed on the seasonal variability of influent concentrations of indicator organisms (Rose et al., 2004). To address this need, influent indicator organism concentrations were plotted over the course of the year and visually analyzed for seasonal trends (Figure 1). Typical influent concentrations for all four indicators varied by approximately one order of magnitude with occasional excursions (Table 1). For all participating WRRFs, both viral and bacterial indicator influent concentrations remained fairly constant over the season. This is similar to observations as reported by Rose et al. (2004), Mandilara et al. (2006), Aw and Gin (2010), and Flannery, Keaveney, Rajko‐Nenow, O'Flaherty, and Doré (2012). The reported values in the literature, however, suggest a broader range of influent coliphage concentrations than observed in this study. For example, Rose et al. (2004) found coliphage varied over three orders of magnitude (10^3^–10^6^ PFU/100 ml) with ATCC host strain 15597 and male‐specific coliphages varying six orders of magnitude (10^2^ to 10^8^ PFU/100 ml) with ATCC host strain 700891. Indicator concentrations may vary depending on the enumeration method and specific WRRF's geographic location, population served, and influent wastewater characteristics.

Concerning the relative concentration of each indicator in relation to the other indicators, the bacterial indicators had higher average influent concentrations than the viral indicators, with *E. coli* having the highest annual average concentration (6.5 ± 0.45 log CFU/100 ml) followed by enterococci (5.8 ± 0.40 log CFU/100 ml), somatic coliphages (5.2 ± 0.49 log PFU/100 ml), and male‐specific coliphages (5.0 ± 0.39 log PFU/100 ml).

### Overall fate and persistence of bacterial and viral indicators

**Figure 2:**
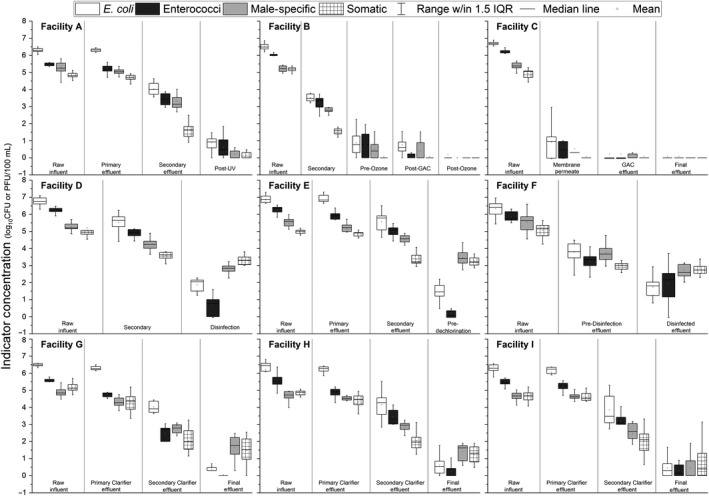
Box‐and‐whisker plots of indicator organism concentrations at each sampling location for WRRFs A through I. Boxplot range is the 25th–75th percentile. Whisker range is 1.5 × the interquartile range (IQR). Means are depicted with squares.

Viral indicator and bacterial indicator concentrations depended on both the treatment process configuration and disinfectant (Figure 2). Mean influent, mean effluent, and mean log reductions for each indicator are reported in Table 1. Bacterial indicator reductions ranged from 4.0 to 6.7 logs, and viral indicator reductions ranged from 1.6 to 5.4 logs. The observed bacterial and viral indicator log reductions for WRRFs A, B, C, and G through I may have underestimated the actual log reductions, as these WRRFs all had typical effluent concentration values at or below the detection limit.

These results are within previous reported log reductions for WRRFs. McMinn, Ashbolt, et al. (2017) compared multiple WRRF studies and concluded that overall fecal indicator bacteria reductions (mean reductions were 2.38 ± 1.26 and 2.22 ± 1.61 for *E. coli* and enterococci, respectively) were greater than coliphages reductions (mean reductions were 1.46 ± 1.18 and 1.46 ± 1.24 for male‐specific and somatic coliphages, respectively). Pouillot et al. (2015) calculated mean male‐specific coliphage log reductions for conventional WRRFs, and these ranged from 2.9 to 4.3 for WRRFs with chlorine and UV disinfection, respectively. Flannery et al. (2012) reported an average mean log reduction of 2.13 for FRNA bacteriophages (male‐specific bacteriophages with an RNA genome) for a conventional activated sludge WRRF without disinfection.

### Indicator reduction through primary treatment

The maximum average primary treatment log reduction for all four indicators was 0.9 (Supporting Information Table S21). Of the indicators, *E. coli* had the lowest mean log reduction in primary processes and enterococci had the highest mean reduction in primary processes. Both somatic coliphage and male‐specific coliphage had a mean log removal between *E. coli* and enterococci. This confirms previous observations that log reductions of up to 0.4 are possible for male‐specific and somatic coliphages through primary treatment (Flannery et al., 2012; Lucena et al., 2004; Mandilara et al., 2006). Flannery et al. (2012) reported similar mean log reductions for primary processes with *E. coli* and FRNA bacteriophages with 0.15 and 0.32 logs, respectively.

**Figure 3:**
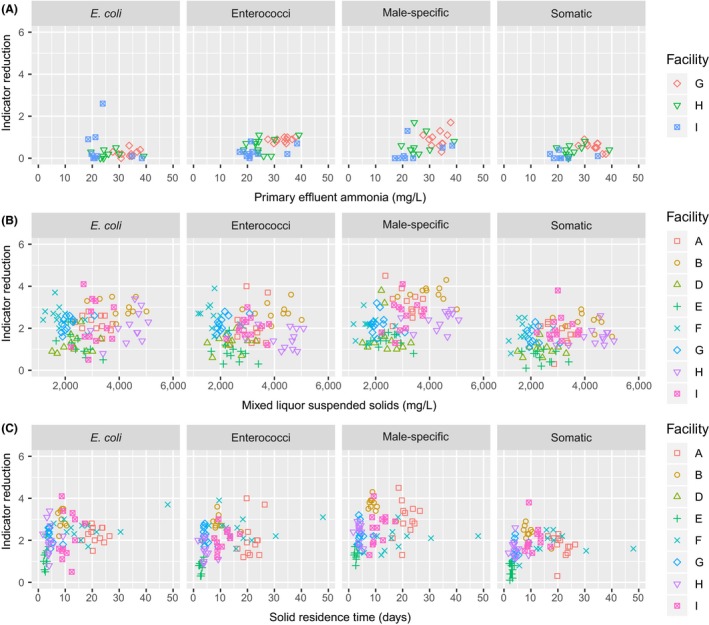
(a) Scatterplots for primary process indicator reduction as a function of primary effluent ammonia (WRRFs G, H, and I); (b) scatterplots for secondary process indicator reduction as a function of the MLSS (All WRRFs except C); and (c) scatterplots for secondary process indicator reduction as a function of the SRT (all WRRFs except C).

Of the process parameters that were explored for indicator reduction, primary effluent ammonia correlated with enterococci (*p* = 8.364e‐05), male‐specific (*p* = 0.0002), and somatic coliphage (*p* = 0.0002) removal through preliminary and primary processes (Figure 3). Primary effluent ammonia did not have a strong relationship with *E. coli* reduction (*p* = 0.5762). Regarding influent TSS and TSS removal, influent TSS correlated with primary process male‐specific (*p* = 0.0126) and somatic coliphage (*p* = 0.0137) reduction; however, no significant correlations were observed with TSS removal and primary process indicator reduction. These observations suggests that WRRFs with higher influent ammonia concentrations may have higher removals of indicators, except for *E. coli*, in primary processes than WRRFs with lower influent concentrations of ammonia. One potential mechanism that explains the relationship with coliphage reduction in primary processes and primary effluent ammonia is work by Oishi et al. (2017) that demonstrated that ammonia inactivates the male‐specific coliphage MS2 through genomic damage. It is possible that enterococci may be more susceptible to ammonia than *E. coli*; however, further work is needed to confirm these observations. Furthermore, primary clarifier overflow rates were not determined in this study. It is likely that primary clarifier overflow rates influence indicator removal, suggesting that this hypothesis should be further evaluated.

### Indicator reduction through secondary treatment

Secondary treatment reductions for the various indicators ranged up to 4 logs and depended on the indicator and secondary process (Figure 2 and Supporting Information Table S22). Previous work by Rose et al. (2004) found similar reductions for secondary processes, noting that bacterial reductions ranged from 1.4 to 3 logs, and somatic coliphage reductions ranged from 0.06 to 3 logs. On average, for secondary processes, the reduction in bacterial indicators was slightly greater than the reduction in viral indicators, but the difference was not significantly different (*p* = 0.4642). WRRFs with BNR processes (WRRFs A F, G, H, and I) had greater coliphage reduction than non‐BNR activated sludge WRRFs (WRRFs D and E; *p* < 2.2e‐16).

Regarding the secondary process parameters that predicted secondary removal, both the MLSS and SRT were correlated with secondary process removal for the indicators (Figure 3). MLSS correlated with secondary process reductions for *E. coli* (*p* = 0.0525), male‐specific coliphages (*p* = 6.095e‐05), and somatic coliphages (*p* = 0.0055). SRT correlated with *E. coli* (*p* = 0.0046), enterococci (*p* = 0.0012), male‐specific (*p* = 0.0108), and somatic coliphage reductions (*p* = 0.0090). Rose et al. (2004) had similar observations suggesting that both higher MLSS concentrations and SRTs tended to increase indicator removal. Higher MLSS concentrations and SRTs both increase time for indicator decay and predation. Work by Pinheiro et al. (2007) suggested that protozoa predation can inactivate coliphages; therefore, it is possible that higher animal predation from protozoa and rotifers may be responsible for additional removal in secondary processes with longer SRTs. Alternatively, many WRRFs with BNR secondary processes operate with lower secondary clarifier overflow rates than non‐BNR WRRFs (Great Lakes, 2014). The lower clarifier overflow rates and longer settling times, which are limited by higher solids loading rates, may be responsible for additional reductions in BNR processes. Taken together, this suggests that WRRFs that operate in a BNR configuration with higher MLSS, higher SRTs, and lower secondary clarifier overflow rates will likely have the highest indicator removal; however, additional work is needed to elucidate and validate the underlying mechanisms of coliphage removal in secondary processes. Future work should include an additional sampling point at the end of the biological treatment before clarification, and future work should evaluate secondary process operation by varying only one of the secondary process parameters while monitoring the others and vice versa.

### Indicator reduction through tertiary treatment

All reported tertiary treatment log reductions (WRRFs B and C) were less than 1.0 log because the observed tertiary influent concentrations were low, limiting the calculated log reduction. The potential log reduction for these tertiary processes is expected to be higher than the observed values, because the observed values were limited by low process influent concentrations and by the volumes of sample (100 ml) analyzed in this study. Potential NPDES coliphage effluent limits are not expected to be a concern for these WRRFs, as the final effluent concentration for coliphages at Facilities B and C were at or below the detection limit.

If tertiary treatment log removal of indigenous indicators is to be determined, future work is needed with additional methods to concentrate larger volumes of water for quantification (e.g., McMinn, Huff, Rhodes, & Korajkic, 2017; Rhodes, Huff, Hamilton, & Jones, 2016; USEPA, 2018), such as 1–10 L. Alternatively, spiking with high stock solutions of indicators is possible for validating process performance. For example, MS2 coliphage is used to estimate log reduction values for microfiltration and ultrafiltration (Amarasiri et al., 2017) and is used to validate UV reactors.

### Indicator reduction through disinfection

**Figure 4:**
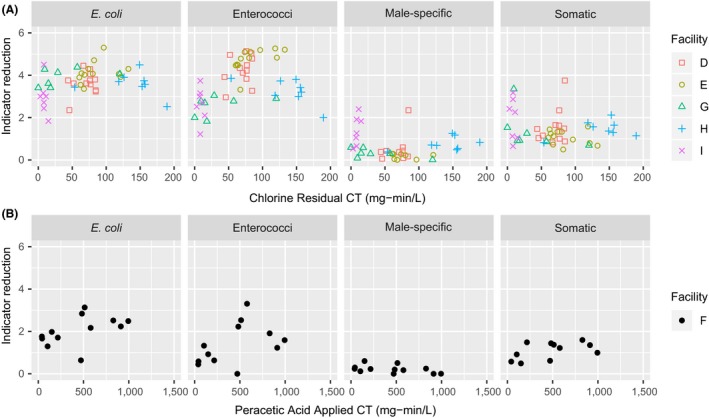
Bacterial and viral indicator disinfection reduction as a function of residual CT for chlorine (WRRFs D, E, G, H, and I) and applied CT PAA (WRRF F).

The indicator reduction depended on both the disinfectant and the indicator for the two UV systems, the one ozone system, the one PAA system, and the five sodium hypochlorite systems (Figures 2 and 4, and Supporting Information Table S23). In general, the disinfectants performed better on the bacterial indicators than on viral indicators; however, in some cases, the influent concentrations limited the observed log reduction.

Sodium hypochlorite disinfection in the presence of ammonia (forming chloramines at typical effluent ammonia concentrations and chlorine doses) provided 3 to 4 logs of reduction for both *E. coli* and enterococci and provided 1 to 2 logs of reduction for somatic coliphages with less than 1 log for male‐specific coliphages. On average, somatic coliphages were more susceptible than male‐specific coliphages to chloramine disinfection. CT values were calculated for each WRRF with chlorine disinfection, and the indicator log reductions were plotted as a function of residual CTs (Figure 4). Based on fixed‐intercept linear models fitted to the data, enterococci were the most sensitive to chloramines followed by *E. coli*, somatic coliphages, and male‐specific coliphages. Previous work has shown the relative resistance of coliphage to combined chlorine (Dunkin et al., 2017; Sobsey, Battigelli, Shin, & Newland, 1998; Tyrrell, Rippey, & Watkins, 1995). The relative resistance of viral indicators to chloramines is problematic, because many WRRFs in the United States use this form of disinfection. In a survey on wastewater disinfection, Leong, Kua, and Tang (2008) found that 71% of the surveyed WRRFs used chlorine for disinfection, and although the exact percentage of WRRFs that completely remove ammonia (nitrify) is not reported, this suggests a portion of the WRRFs in the United States use a disinfection process (chlorine in the presence of ammonia) that slowly disinfects viral indicators when compared to traditional bacterial indicators.

In contrast to combined chlorine kinetics, free chlorine provides a rapid reduction in coliphages and viruses (Cromeans, Kahler, & Hill, 2010; Munakata et al., 2011; Soroushian, Erdal, Shyamasundar, & Tchobanoglous, 2010). In wastewater, however, free chlorine disinfection is not common due to the challenges associated with breakpoint chlorination. In addition, WRRFs using chlorine generally do not measure free chlorine residual, and are only required to measure total residual chlorine, so there are limited data on the prevalence of free residual chlorine in wastewater disinfection.

The low pressure‐UV (LP‐UV) WRRF provided greater than 2 logs of inactivation for *E. coli*, enterococci, and somatic coliphages; however, these log inactivation values were limited by the low pre‐disinfection microbial concentrations. The LP‐UV log reduction in male‐specific coliphages was 1.5 log, and this was also limited by the pre‐disinfection coliphage concentration. Due to the presence of multiple nondetects in the LP‐UV effluent, disinfection kinetics and sensitivity values for indigenous somatic and male‐specific coliphages could not be determined in this study. Reported sensitivity values for the male‐specific coliphage MS2 are 20 mJ/cm^2^/log inactivation (Hijnen, Beerendonk, & Medema, 2006), and reported values for T1UV and T1, both somatic coliphages, are 5 and 2.5 mJ/cm^2^/log inactivation, respectively (Hargy, Lawal, Bemus, Townsend, & Sobrinho, 2008; Stefan, Odegaard, Petri, Rowntree, & Sealey, 2007).

The ozone WRRF provided greater than 2 logs of reduction for *E. coli*, enterococci, and somatic coliphages; however, these values were limited by the pre‐disinfection concentrations. Similar to UV, the observed ozone log reduction in male‐specific coliphages was 1.4, and the reported log reduction was limited by the low pre‐disinfection coliphage concentration. Typical reported log removals for CTs of less than 4 mg‐min/L provide between 3 and 6 logs of removal with indigenous and laboratory male‐specific and somatic coliphages (Shin & Sobsey, 2003; Tyrrell et al., 1995).

The effectiveness of PAA on indicator organisms for WRRF F, at typical PAA doses and contact times, was as follows: *E. col*i (2.1 log reduction) > enterococci (1.4 log reduction) > somatic coliphages (1 log reduction) > male‐specific coliphages (0.1 log reduction). For the PAA analysis, indicator log reduction results were plotted as a function of the applied PAA CT. These results are in agreement with previous studies that showed higher inactivation for bacterial indicators than viral indicators with PAA (Kitis, 2004; Zanetti, De Luca, Sacchetti, & Stampi, 2007). Although the indicator removals reported here were lower than chlorine (with and without ammonia), the dose of PAA used at this WRRF is low (typically less than 2 ppm). Others have demonstrated the effectiveness of PAA for coliphage reduction at higher doses (typically between 5 and 15 mg/L) and contact times (typically greater than 20 min) for 1 log reduction (Koivunen, and Heinonen‐Tanski, 2005; Gehr, Wagner, Veerasubramanian, & Payment, 2003; Park, Lee, Bisesi, & Lee, 2014).

Unfortunately, coliphage RWQC may limit the adoption of PAA, which is considered a green disinfectant. For example, PAA does not form chlorinated disinfection byproducts, it does not increase the salt burden on freshwater receiving streams, it breaks down into a biodegradable carbon source, and it increases the dissolved oxygen content of the water. Interestingly, with PAA, Dunkin et al. (2017) reported higher reductions with murine norovirus than with coliphages, which highlights the conservativeness of coliphages with respect to some viruses and suggests that further work is needed to evaluate indicator susceptibility versus pathogen susceptibility.

### Coliphages as indicators of enteric viruses

Figure 1 includes seasonal enteric virus influent data over the course of 1 year for WRRFs G, H, and I. Occurrence and variability of enteric viruses in raw influent corroborate other studies quantifying wastewater noroviruses and adenoviruses over similar time periods (e.g., Carducci & Verani, 2013; Eftim et al., 2017; Grøndahl‐Rosado, Yarovitsyna, Trettenes, Myrmel, & Robertson, 2014; Hata, Kitajima, & Katayama, 2012; Kitajima, Iker, Pepper, & Gerba, 2014). Compared to the bacterial and viral indicators, the enteric viruses showed greater variability over the course of the year, similar to the higher enteric virus variability as reported by Grøndahl‐Rosado et al. (2014). The greater variability in molecular‐based pathogen data may be an artifact of the error rates in scaling up quantitative PCR data from concentrations per reaction to concentrations per liter, the different study concentration and extraction methodologies used, and/or the prevalence of disease in the sewershed population. In addition, no obvious enteric virus seasonal trends were observed in this data set as similar to Qiu et al. (2015), Kitajima et al. (2014), and Kuo et al. (2010). This is in contrast to work reported by Katayama et al. (2008) and Pérez‐Sautu et al. (2012), which suggested that norovirus concentrations were higher in the winter months. More work on longer time series observations for viruses and indicators is recommended to establish seasonal trends.

**Figure 5:**
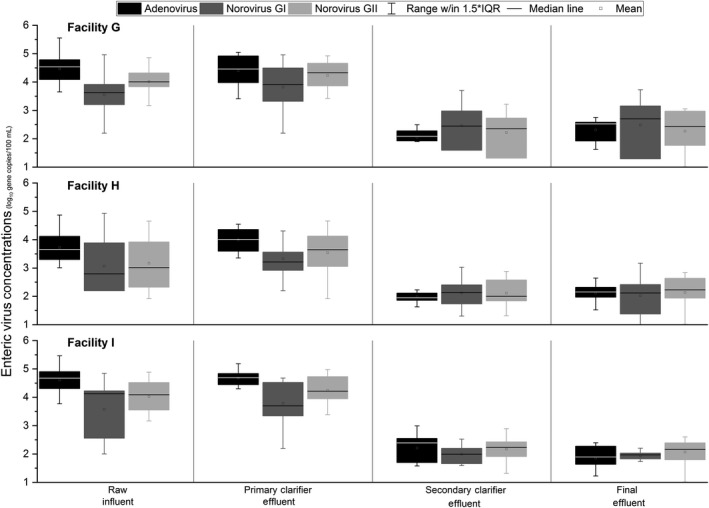
Box‐and‐whisker plots of enteric virus concentration (gene copies) at each sampling location for Facilities G through I. Boxplot range is the 25th–75th percentile. Whisker range is 1.5 × the interquartile range (IQR). Means are depicted with squares.

Figure 5 shows boxplots of enteric virus concentrations over the course of each treatment step for WRRFs G, H, and I. Raw influent concentrations (mean ± *SD*) for these enteric viruses (WRRF G adenovirus: 4.5 ± 0.54, norovirus GI: 3.5 ± 0.81, norovirus GII: 4.0 ± 0.46; WRRF H adenovirus: 3.7 ± 0.56, norovirus GI: 3.1 ± 0.98, norovirus GII: 3.2 ± 0.96; WRRF I adenovirus: 4.6 ± 0.47, norovirus GI: 3.6 ± 0.97, norovirus GII: 4.0 ± 0.58) were closer to coliphage concentrations (see Tables 1 and 2) than fecal indicator bacteria concentrations. Adenovirus had the highest mean influent concentration and frequency of detection (93% of the total samples—134/144). Norovirus GII (90% total frequency of detection—129/144) had higher mean concentrations than norovirus GI (83% total frequency of detection—119/144) at all three WRRFs. Boxplots of enteric viruses across the different treatment steps ranged between 1 and 3 logs, which were greater than the variability seen in boxplots of bacterial and viral indicators for the same WRRFs (Figure 2).

**Table 2:** Mean log reduction of enteric pathogens

Enteric virus	Whole process		1° + 2° Treatment		Chlorination	
	Mean	*SD*	Mean	*SD*	Mean	*SD*
WRRF G						
Adenovirus	2.15	0.37	2.4	0.45	−0.25	0.35
Norovirus GI	0.93	0.62	0.95	0.57	−0.02	0.24
Norovirus GII	1.67	0.63	1.72	0.61	−0.05	0.33
WRRF H						
Adenovirus	1.6	0.7	1.74	0.79	−0.14	0.39
Norovirus GI	1.05	0.71	0.95	0.64	0.1	0.36
Norovirus GII	1.03	0.72	1.06	0.74	−0.03	0.36
WRRF I						
Adenovirus	2.75	0.74	2.39	0.81	0.35	0.5
Norovirus GI	1.63	0.94	1.58	0.84	0.05	0.3
Norovirus GII	1.95	0.49	1.85	0.46	0.1	0.26

Table 2 shows the mean log reductions in enteric virus concentrations. Whole treatment process enteric virus log reductions varied from 0.93 to 2.75 logs depending on the enteric virus and WRRF. Even though accurate comparisons are difficult due to differences in WRRF processes, the enteric virus pathogen log reductions were similar to the removal rates seen in other studies (Carducci, Battistini, Rovini, & Verani, 2009; Carducci & Verani, 2013; Hewitt, Leonard, Greening, & Lewis, 2011; Katayama et al., 2008; Petrinca et al., 2009; Wen, Tutuka, Keegan, & Jin, 2009). The two WRRFs with higher levels of secondary treatment (WRRFs G & I) had larger log removals than WRRF H; this finding is similar to what was documented in other WRRF comparisons (Rose et al., 2004; Schmitz, Kitajima, Campillo, Gerba, & Pepper, 2016). The total log reductions in enteric viruses over the entire course of the treatment process were more similar to the 2–3 log reduction seen for infectious coliphage indicators than the 3–4 log reduction seen for cultured fecal indicator bacteria, similar to findings described in Dias, Ebdon, and Taylor (2018), McMinn, Ashbolt, et al. (2017), Hata et al. (2012), and Rose et al. (2004). This suggests that viral indicators are more “ideal” indicators for viral pathogen reduction at WRRFs than the traditional bacterial indicators (Dias et al., 2018). It should be noted that the enteric virus log reductions documented here are conservative since PCR was used for quantification. PCR‐based methods overestimate infectious viruses due to the detection of extracellular nucleic acids and noninfectious virus particles.

Future work is needed to determine pathogen abundance using a combination of molecular‐ and culture‐based methods. While molecular methods overestimate viable pathogens, culture‐based quantifications of enteric viruses may underestimate their abundance (Gerba, Betancourt, & Kitajima, 2017). Newer technologies, for example, propidium monoazide (PMA)‐based molecular methods, hold promise for detecting more reliably the “true” abundance of viable pathogens (Kim & Ko, 2012; Li & Chen, 2013; Li et al., 2014; Randazzo et al., 2018).

Associations in raw influent (*N* = 42) and final effluent (*N* = 41) between enteric viruses and fecal indicators were assessed using Spearman's correlations (Supporting Information Table S24). Correlations that were significant can be seen in bold font. Adenovirus was not significantly correlated to any cultured indicator or the two noroviruses. Norovirus GI and GII have significant negative associations with *E. coli*, while norovirus GII had significant negative associations with enterococci and positive associations with adenovirus in raw influent samples. In the raw influent samples, the only significant coliphage correlation was the positive association between somatic coliphage and enterococci. Norovirus GI and GII had a significant positive correlation to both of the monitored viral indicators and a significant negative correlation to *E. coli* in final effluent samples. Male‐specific coliphage had significant correlations with the other indicators (positive with somatic coliphage and negative with the bacterial indicators). Conflicting associations in different wastewater steps, as seen in here between raw influent and final effluent samples, is noted by others across environmental matrices (Grøndahl‐Rosado et al., 2014; Ottoson et al., 2006; United States Environmental Protection Agency, 2015; Wu, Long, Das, & Dorner, 2011). No single fecal indicator (bacteria or virus) correlated positively with the three enteric viruses. The continued sampling and analysis of enteric viruses and fecal indicators by both infectivity methods and molecular PCR‐based methods is needed to develop a more robust and long‐term database to reliably predict enteric pathogen presence (Wu et al., 2011).

### WRRF process factors influencing coliphage reduction and treatment implications

Key conclusions and observations are as follows: WRRFs with BNR secondary processes had greater indicator organism reduction in the secondary process (typically greater than 2 logs), which, in turn, provided a higher quality effluent that was more amenable to disinfection. Facilities with non‐BNR activated sludge and chlorine (de facto chloramine) disinfection had approximately 5 logs of reduction for bacterial indicators; however, these WRRFs reduced viral indicator concentrations by only 1–3 logs. Facilities in this study with UV had a high‐quality secondary effluent, which contained lower concentrations of all four indicators. The WRRFs with UV and BNR had overall log reductions of greater than 4 logs for both the bacterial indicators and viral indicators. The ozone WRRF with BNR in this study had a high‐quality secondary effluent and was able to achieve greater than 5 logs of reduction for each indicator organism. The WRRF with PAA achieved 4 logs of reduction for the bacterial indicators and approximately 2–3 logs of reduction for the viral indicators.

Recent trends to increase ammonia removal have shifted more WRRFs to BNR processes, which will likely increase viral indicator removal and also make the secondary effluent more amenable to disinfection. Given the challenges associated with achieving breakpoint chlorination (free chlorine disinfection) in wastewater, free chlorine disinfection may not be a feasible alternative to improve viral reduction for WRRFs except where the effluent ammonia concentration is very low, suggesting coliphage criteria could shift disinfection strategies toward UV. Alternately, some WRRFs that do not have space available for BNR processes may move toward MBRs, which have greater indicator reduction than conventional treatment (Facility C and Zhang & Farahbakhsh, 2007).

## Conclusions

Although there is an active scientific debate on the epidemiological relationships between indicators, enteric viruses, and the human health risks from waterborne disease, the work herein suggests that viral indicators correlate better with the fate of enteric viruses as estimated by their nucleic acids than bacterial indicators. In addition, this work suggests that many WRRFs in the United States (especially those using chlorine disinfection with ammonia in the effluent or those using PAA) have greater bacterial indicator reduction than viral indicator reduction. This suggests that viral indicator reduction is variable between WRRFs, and one logical assumption is that viral indicators may serve as better predictors of the fate of enteric viruses. Given these considerations, it is possible that coliphage RWQC could improve viral reduction in WRRFs and thus reduce point source loading of enteric viruses to the environment, depending on the numeric criteria established for coliphages and on how coliphage numeric criteria affect NPDES permit limits for WRRFs. Future work should evaluate the impact of WRRF effluent on illness risks in recreational water compared to illness risks from wastewater collection system overflows, other environmental sources of fecal contamination, person‐to‐person contact, and fomites. Additional work should also estimate the recommended concentration of viral indicators to provide an acceptable level of illness risk that balances the funds available for treatment given the multiple treatment objectives that WRRFs face.
