# Supplementary material for: Evaluating the fate of bacterial indicators, viral indicators, and viruses in water resource recovery facilities
Source: Water Environ Res. 2019 Apr 20;91(9):830–42. doi: 10.1002/wer.1096 (PMC6849880; doi:10.1002/wer.1096)
Supplement: Supplementary file 1 [file WER-91-830-s001.docx]

**EVALUATING THE FATE OF BACTERIAL INDICATORS, VIRAL INDICATORS, AND VIRUSES IN WATER RESOURCE RECOVERY FACILITIES**

**SUPPORTING INFORMATION**

**ADDITIONAL FIGURES AND TABLES**

Thomas Worley-Morse^1*^, Melanie Mann^2^, Wendell Khunjar^3^, Lola Olabode^4^, and Raul Gonzalez^5^

^1^Hazen and Sawyer, Lakewood, Colorado

^2^Hazen and Sawyer, Raleigh, North Carolina

^3^Hazen and Sawyer, Fairfax, Virginia

^4^Water Research Foundation, Alexandria, Virginia

^5^Hampton Roads Sanitation District, Virginia Beach, Virginia

**Table S1.** Facility Characteristics, Process Configurations, and Sampling Locations

| **Facility Name** | **Process Configuration** | **Sampling Location A** | **Sampling Location B** | **Sampling Location C** | **Sampling Location D** | **Sampling Location E** |
| --- | --- | --- | --- | --- | --- | --- |
| Facility A | 5-Stage BNR with LPHO UV | Raw influent | Primary effluent | Secondary effluent | Post UV | N/A |
| Facility B | 5-Stage BNR with tertiary clarification (ferric), dual train filtration (deep bed and UF GAC/BAC), and ozone | Raw influent | Secondary effluent | Pre-ozone effluent | Post GAC | Post-ozone effluent |
| Facility C | 5-Stage BNR with MBR and MP UV | Raw influent | Membrane permeate | Post-GAC (carbon effluent) | Post UV | N/A |
| Facility D | Aerated grit (no primary clarifiers) with step-aeration activated sludge and sodium hypochlorite | Raw influent | Secondary effluent | Disinfected effluent | N/A | N/A |
| Facility E | Pure oxygen activated sludge with sodium hypochlorite | Raw influent | Primary effluent | Secondary effluent | Pre-dechlorination | N/A |
| Facility F | Sequencing batch reactor with peracetic acid | Raw influent | Pre-disinfected effluent | Post-disinfected effluent | N/A | N/A |
| Facility G | Integrated fixed film activated sludge with sodium hypochlorite | Raw influent | Primary effluent | Secondary effluent | Post-dechlorination | N/A |
| Facility H | Three cell activated sludge with swing/anoxic aerobic for the first cell with sodium hypochlorite | Raw influent | Primary effluent | Secondary effluent | Disinfected effluent | N/A |
| Facility I | 3-Stage BNR (A2O) with sodium hypochlorite | Raw influent | Primary effluent | Secondary effluent | Disinfected effluent | N/A |

**Table S2. Primers Used for Molecular Methods**

|  | **Forward** | **Reverse** |  | **Detection Limit (DL)** |  |
| --- | --- | --- | --- | --- | --- |
| **Virus** | **Primer** | **Primer** | **Probe** | **copies/rxn** | **Reference(s)** |
| Adenovirus | GGACGCCTCGGAGTACCTGAG | ACIGTGGGGTTTCTGAACTTGTT | FAM-CTGGTGCAGTTCGCCCGTGCCA-BHQ | 5.03 | Jothikumar et al., 2005 |
| Norovirus GI | CGCTGGATGCGNTTCCAT | CCTTAGACGCCATCATCATTTAC | FAM-TGGACAGGAGAYCGCRATCT-TAMRA | 3.13 | Da Silva et al., 2007 |
| Norovirus GII | ATGTTCAGRTGGATGAGRTTCTCWGA | TCGACGCCATCTTCATTCACA | FAM-AGCACGTGGGAGGGCGATCG-BHQ | 1.65 | Butot et al., 2010; Da Silva et al., 2007 |
| **Internal Control** |  |  |  |  |  |
| Hepatitis G | CGGCCAAAAGGTGGTGGATG | CGACGAGCCTGACGTCGGG | FAM-AGGTCCCTCTGGCGCTTGTGGCGAG-TAMRA | . | Cashdollar et al., 2013 |
| Sketa | GGTTTCCGCAGCTGGG | CCGAGCCGTCCTGGTCTA | FAM-AGTCGCAGGCGGCCACCGT-BHQ1 | . | Haugland et al., 2005 |

**Table S3. Bacterial Indicator Concentrations for Facility A**

| **Facility A** | ***E. coli* (CFU/100 mL)** | | | | **Enterococci (CFU/100 mL)** | | | |
| --- | --- | --- | --- | --- | --- | --- | --- | --- |
| **Date** | **Raw Influent** | **Primary Effluent** | **Secondary Effluent** | **Post UV** | **Raw Influent** | **Primary Effluent** | **Secondary Effluent** | **Post UV** |
| Month 1 (May)^2^ | 2.3E+06 | 2.3E+06 | 3.6E+03 | 9.0E+00 | 1.3E+06 | 1.4E+05 | 1.8E+03 | 9.0E+00^1^ |
| Month 2 (June) | 1.1E+06 | 2.0E+06 | 9.6E+03 | 1.0E+00^1^ | 6.3E+04 | 5.1E+04 | 3.4E+03 | 1.0E+00^1^ |
| Month 3 (July) | 2.4E+06 | 2.1E+06 | 4.9E+03 | 9.0E+00 | 3.7E+05 | 2.3E+05 | 9.0E+02 | 2.0E+00 |
| Month 4 (August) | 1.8E+06 | 2.2E+06 | 2.3E+04 | 1.6E+01 | 3.0E+05 | 1.1E+05 | 3.0E+03 | 3.0E+00 |
| Month 5 (September) | 9.0E+05 | 1.5E+06 | 5.8E+03 | 8.0E+00 | 3.2E+05 | 1.5E+05 | 1.6E+01 | 1.0E+00^1^ |
| Month 6 (October) | 1.6E+06 | 1.8E+06 | 1.0E+04 | 3.0E+01 | 2.3E+05 | 1.7E+05 | 3.3E+01 | 1.0E+00^1^ |
| Month 7 (November)^3^ | 1.8E+06 | 2.8E+06 | 2.0E+04 | 4.0E+00 | 3.4E+05 | 4.0E+05 | 7.0E+03 | 1.6E+01 |
| Month 8 (December) | 2.5E+06 | 1.9E+06 | 2.2E+04 | 1.7E+02 | 2.7E+05 | 1.1E+05 | 5.6E+03 | 6.8E+01 |
| Month 9 (January) | 1.7E+06 | 1.5E+06 | 3.7E+03 | 1.0E+00 | 2.9E+05 | 2.5E+05 | 2.3E+03 | 2.0E+00 |
| Month 10 (February) | 3.2E+06 | 2.4E+06 | 6.4E+03 | 5.0E+00 | 3.2E+05 | 2.4E+05 | 2.5E+03 | 6.0E+00 |
| Month 11 (March)^2^ | 2.0E+06 | 1.5E+06 | 4.3E+04 | 3.7E+00 | 7.2E+04 | 1.7E+05 | 7.4E+03 | 2.8E+00 |
| Month 12 (April) | 2.5E+06 | 2.6E+06 | 2.4E+04 | 1.0E+01 | 3.8E+05 | 1.3E+05 | 5.8E+03 | 1.4E+01 |
|  |  |  |  |  |  |  |  |  |
| ^1^ Below limit of detection | |  |  |  |  |  |  |  |
| ^2^ Warm Sample |  |  |  |  |  |  |  |  |
| ^3^ Greater than 48-hour hold time | |  |  |  |  |  |  |  |

**Table S4. Viral Indicator Concentrations for Facility A**

| **Facility A** | **Male specific (PFU/100 mL)** | | | | **Somatic (PFU/100 mL)** | | | |
| --- | --- | --- | --- | --- | --- | --- | --- | --- |
| **Date** | **Raw Influent** | **Primary Effluent** | **Secondary Effluent** | **Post UV** | **Raw Influent** | **Primary Effluent** | **Secondary Effluent** | **Post UV** |
| Month 1 (May)^2^ | 6.0E+04 | 5.9E+04 | 2.7E+03 | 1.0E+00^1^ | 1.9E+05 | 9.9E+04 | 5.3E+04 | 1.0E+00^1^ |
| Month 2 (June) | 5.4E+04 | 3.4E+04 | 1.0E+00^1^ | 1.0E+00^1^ | 1.6E+05 | 1.2E+05 | 8.5E+02 | 1.0E+00^1^ |
| Month 3 (July) | 1.0E+05 | 8.5E+04 | 3.1E+01 | 2.0E+00 | 6.4E+05 | 1.1E+05 | 4.9E+02 | 4.0E+00 |
| Month 4 (August) | 1.3E+05 | 4.2E+04 | 3.1E+02 | 1.0E+00^1^ | 2.3E+05 | 1.1E+05 | 7.7E+02 | 1.0E+00^1^ |
| Month 5 (September) | 1.3E+05 | 6.9E+04 | 8.0E+00 | 2.0E+00 | 4.2E+05 | 3.4E+05 | 5.4E+03 | 3.0E+00 |
| Month 6 (October) | 6.9E+04 | 4.1E+04 | 1.5E+01 | 1.0E+00^1^ | 1.6E+05 | 1.3E+05 | 2.2E+03 | 2.0E+00 |
| Month 7 (November)^3^ | 7.4E+04 | 2.1E+04 | 6.0E+01 | 1.0E+00^1^ | 1.6E+05 | 2.3E+05 | 1.0E+04 | 1.0E+00^1^ |
| Month 8 (December) | 3.3E+04 | 4.4E+04 | 6.4E+01 | 3.0E+00 | 8.7E+04 | 7.7E+04 | 1.6E+03 | 1.3E+01 |
| Month 9 (January) | 6.4E+04 | 5.9E+04 | 7.2E+01 | 1.0E+00^1^ | 2.6E+04 | 3.6E+04 | 1.5E+03 | 1.0E+00^1^ |
| Month 10 (February) | 6.5E+04 | 7.0E+04 | 2.1E+01 | 1.0E+00 | 3.1E+04 | 5.3E+04 | 9.5E+02 | 1.0E+00^1^ |
| Month 11 (March)^2^ | 2.1E+04 | 3.7E+04 | 6.2E+01 | 1.0E+00^1^ | 2.8E+06 | 1.5E+05 | 1.3E+03 | 1.0E+00^1^ |
| Month 12 (April) | 5.6E+04 | 6.4E+04 | 3.0E+01 | 3.0E+00 | 2.8E+05 | 1.0E+05 | 1.0E+03 | 1.0E+00^1^ |
|  |  |  |  |  |  |  |  |  |
| ^1^ Below limit of detection | |  |  |  |  |  |  |  |
| ^2^ Warm Sample |  |  |  |  |  |  |  |  |
| ^3^ Greater than 48-hour hold time | |  |  |  |  |  |  |  |

**Table S5. Bacterial Indicator Concentrations for Facility B**

| **Facility B** | ***E. coli* (CFU/100 mL)** | | | | | **Enterococci (CFU/100mL)** | | | | |
| --- | --- | --- | --- | --- | --- | --- | --- | --- | --- | --- |
| **Date** | **Raw** | **Secondary** | **Pre-Ozone** | **Post GAC** | **Post Ozone** | **Raw** | **Secondary** | **Pre-Ozone** | **Post GAC** | **Post Ozone** |
| Month 1 (May) | 3.7E+06 | 2.4E+04 | 1.8E+02 | 4.5E+02 | 1.6E+02 | 6.3E+05 | 5.4E+03 | 9.0E+01 | 9.0E+01 | 2.2E+01 |
| Month 2 (June)^2^ | 5.0E+06 | 2.5E+03 | 5.0E+00 | 1.0E+01 | 1.0E+00^1^ | 1.1E+06 | 1.4E+03 | 1.0E+00 | 1.0E+00 | 1.0E+00^1^ |
| Month 3 (July)^2^ | 7.2E+06 | 2.6E+03 | 1.0E+01 | 7.0E+00 | 1.0E+00^1^ | 1.0E+06 | 2.2E+03 | 1.0E+00^1^ | 1.0E+00^1^ | 1.0E+00 |
| Month 4 (August)^2^ | 1.6E+06 | 4.5E+02 | 1.0E+00 | 3.5E+01 | 1.0E+00^1^ | 9.7E+05 | 6.3E+02 | 1.0E+00 | 1.0E+00 | 1.0E+00^1^ |
| Month 5 (September) | 3.4E+06 | 6.0E+03 | 1.0E+00 | 5.0E+00 | 1.0E+00^1^ | 1.1E+06 | 2.6E+03 | 1.0E+00^1^ | 2.0E+00 | 1.0E+00^1^ |
| Month 6 (October) | 5.8E+06 | 1.8E+03 | 1.0E+00 | 1.0E+00^1^ | 1.0E+00^1^ | 1.2E+06 | 2.7E+02 | 2.4E+01 | 1.0E+00^1^ | 1.0E+00^1^ |
| Month 7 (November) | 2.7E+06 | 5.1E+03 | 1.5E+01 | 4.0E+00 | 1.0E+00^1^ | 1.5E+06 | 3.1E+03 | 6.0E+00 | 1.0E+00^1^ | 1.0E+00^1^ |
| Month 8 (December) | 2.7E+06 | 2.6E+03 | 6.2E+01 | 3.0E+00 | 1.0E+00^1^ | 1.2E+06 | 1.6E+03 | 7.3E+01 | 1.0E+00^1^ | 1.0E+00^1^ |
| Month 9 (January)^2,3^ | 2.2E+06 | 3.7E+03 | 2.4E+01 | 3.0E+00 | 1.0E+00^1^ | 9.3E+05 | 4.0E+03 | 2.5E+01 | 1.0E+00^1^ | 1.0E+00^1^ |
| Month 10 (February) | 2.2E+06 | 3.4E+03 | 7.0E+00 | 1.0E+00 | 1.0E+00^1^ | 1.0E+06 | 2.2E+03 | 9.0E+00 | 1.4E+00 | 1.0E+00^1^ |
| Month 11 (March) | 2.5E+06 | 5.8E+03 | 4.0E+00 | 2.0E+00 | 1.0E+00 | 9.3E+05 | 3.0E+03 | 1.0E+00 | 1.0E+00 | 1.0E+00 |
| Month 12 (April) | 3.5E+06 | 1.6E+03 | 5.0E+00 | 4.0E+00 | 1.0E+00 | 1.1E+06 | 4.5E+02 | 6.0E+00 | 4.0E+00 | 1.0E+00 |
|  |  |  |  |  |  |  |  |  |  |  |
| ^1^ Below limit of detection | |  |  |  |  |  |  |  |  |  |
| ^2^ Warm Sample |  |  |  |  |  |  |  |  |  |  |
| ^3^ Greater than 48-hour hold time | |  |  |  |  |  |  |  |  |  |

**Table S6. Viral Indicator Concentrations for Facility B**

| **Facility B** | **Male specific (PFU/100 mL)** | | | | | **Somatic (PFU/100 mL)** | | | | |
| --- | --- | --- | --- | --- | --- | --- | --- | --- | --- | --- |
| **Date** | **Raw** | **Secondary** | **Pre-Ozone** | **Post GAC** | **Post Ozone** | **Raw** | **Secondary** | **Pre-Ozone** | **Post GAC** | **Post Ozone** |
| Month 1 (May) | 2.5E+05 | 3.6E+01 | 9.0E+00^1^ | 1.0E+00^1^ | 1.0E+00^1^ | 2.6E+05 | 4.9E+02 | 9.0E+00 | 3.3E+01 | 1.0E+00^1^ |
| Month 2 (June)^2^ | 1.5E+05 | 4.3E+01 | 1.0E+00^1^ | 1.0E+00^1^ | 1.0E+00^1^ | 2.7E+05 | 9.0E+02 | 6.0E+00 | 4.0E+00 | 1.0E+00^1^ |
| Month 3 (July)^2^ | 1.9E+05 | 4.3E+01 | 1.0E+00^1^ | 1.0E+00^1^ | 1.0E+00^1^ | 1.0E+05 | 8.2E+02 | 1.0E+00 | 1.0E+01 | 1.0E+00^1^ |
| Month 4 (August)^2^ | 1.3E+05 | 2.2E+01 | 1.0E+00^1^ | 2.0E+00 | 1.0E+00^1^ | 1.5E+05 | 6.5E+02 | 2.0E+00 | 6.0E+00 | 1.0E+00^1^ |
| Month 5 (September) | 1.2E+05 | 1.6E+01 | 1.0E+00^1^ | 3.0E+00 | 1.0E+00^1^ | 2.6E+05 | 3.0E+02 | 1.0E+00^1^ | 1.1E+01 | 1.0E+00^1^ |
| Month 6 (October) | 8.1E+04 | 3.9E+00 | 1.0E+00^1^ | 1.0E+00^1^ | 1.0E+00^1^ | 1.1E+05 | 5.0E+02 | 1.0E+00^1^ | 1.0E+00^1^ | 1.0E+00^1^ |
| Month 7 (November) | 1.7E+05 | 4.7E+01 | 1.0E+00^1^ | 1.0E+00^1^ | 1.0E+00^1^ | 2.2E+05 | 8.0E+02 | 1.0E+00^1^ | 1.0E+00^1^ | 1.0E+00^1^ |
| Month 8 (December) | 2.3E+05 | 2.9E+01 | 1.0E+00^1^ | 1.0E+00^1^ | 1.0E+00^1^ | 2.0E+05 | 3.9E+03 | 3.4E+01 | 1.0E+00^1^ | 1.0E+00^1^ |
| Month 9 (January)^2,3^ | 1.4E+05 | 1.5E+02 | 1.0E+00^1^ | 1.0E+00^1^ | 1.0E+00^1^ | 8.8E+04 | 2.3E+03 | 4.0E+00 | 1.0E+00^1^ | 1.0E+00^1^ |
| Month 10 (February) | 1.3E+05 | 5.0E+01 | 1.0E+00^1^ | 1.0E+00^1^ | 1.0E+00^1^ | 2.0E+05 | 5.8E+02 | 6.0E+00 | 1.0E+00^1^ | 1.0E+00^1^ |
| Month 11 (March) | 1.7E+05 | 6.3E+01 | 1.0E+00 | 1.0E+00 | 1.0E+00 | 1.2E+05 | 5.2E+02 | 2.0E+00 | 1.0E+00 | 1.0E+00 |
| Month 12 (April) | 1.8E+05 | 3.5E+01 | 1.0E+00 | 1.0E+00 | 1.0E+00 | 1.2E+05 | 5.5E+02 | 3.0E+00 | 1.0E+00 | 1.0E+00 |
|  |  |  |  |  |  |  |  |  |  |  |
| ^1^ Below limit of detection | |  |  |  |  |  |  |  |  |  |
| ^2^ Warm Sample |  |  |  |  |  |  |  |  |  |  |
| ^3^ Greater than 48-hour hold time | |  |  |  |  |  |  |  |  |  |

**Table S7. Bacterial Indicator Concentrations for Facility C**

| **Facility C** | ***E. coli* (CFU/100 mL)** | | | | **Enterococci (CFU/100 mL)** | | | |
| --- | --- | --- | --- | --- | --- | --- | --- | --- |
| **Date** | **Raw Influent** | **Membrane Permeate** | **GAC Effluent** | **Final Effluent** | **Raw Influent** | **Membrane Permeate** | **GAC Effluent** | **Final Effluent** |
| Month 1 (May) | 4.3E+06 | 9.0E+02^1^ | 9.0E+02^1^ | 9.0E+00^1^ | 1.3E+06 | 1.0E+00^1^ | 9.0E+02^1^ | 9.0E+00^1^ |
| Month 2 (June) | 3.5E+06 | 9.0E+00^1^ | 9.0E-01^1^ | 1.0E+00^1^ | 1.7E+06 | 9.0E+00^1^ | 9.8E-01^1^ | 1.0E+00^1^ |
| Month 3 (July) | 4.3E+07 | 9.0E+02^1^ | 1.0E+00^1^ | 1.0E+00^1^ | 1.8E+06 | 9.0E+02^1^ | 1.0E+00^1^ | 1.0E+00^1^ |
| Month 4 (August) | 4.9E+06 | 1.0E+00^1^ | 1.0E+00^1^ | 1.0E+00^1^ | 1.4E+06 | 1.0E+00^1^ | 1.0E+00^1^ | 1.0E+00^1^ |
| Month 5 (September) | 4.3E+06 | 1.0E+00^1^ | 1.0E+00^1^ | 1.0E+00^1^ | 1.6E+06 | 1.0E+00^1^ | 1.0E+00^1^ | 1.0E+00^1^ |
| Month 6 (October)^2^ | 5.9E+06 | 2.8E+01^1^ | 9.0E-01^1^ | 1.0E+00^1^ | 1.4E+06 | 9.0E+00^1^ | 9.0E-01^1^ | 1.0E+00^1^ |
| Month 7 (November) | 4.7E+06 | 9.0E+00^1^ | 9.0E-01^1^ | 1.0E+00^1^ | 1.5E+06 | 9.0E+00^1^ | 9.0E-01^1^ | 1.0E+00^1^ |
| Month 8 (December) | 7.7E+06 | 9.0E+00^1^ | 9.1E-01 | 1.0E+00^1^ | 2.8E+06 | 9.0E+00^1^ | 9.0E-01^1^ | 1.0E+00^1^ |
| Month 9 (January)^3^ | 4.5E+06 | 1.0E+01^1^ | 9.0E-01^1^ | 1.0E+00^1^ | 1.6E+06 | 1.0E+01^1^ | 9.5E-01^1^ | 1.0E+00^1^ |
| Month 10 (February) | 5.5E+06 | 9.0E-01^1^ | 9.0E-01^1^ | 1.0E+00^1^ | 2.2E+06 | 9.0E-01^1^ | 1.0E+00^1^ | 1.0E+00^1^ |
| Month 11 (March) | 2.7E+06 | 9.0E-01^1^ | 9.0E-01^1^ | 1.0E+00^1^ | 8.2E+05 | 9.0E-01^1^ | 1.0E+00^1^ | 1.0E+00^1^ |
| Month 12 (April) | 5.4E+06 | 9.0E-01^1^ | 9.0E-01^1^ | 1.0E+00^1^ | 1.9E+06 | 9.0E-01^1^ | 9.0E-01^1^ | 1.0E+00^1^ |
|  |  |  |  |  |  |  |  |  |
| ^1^ Below limit of detection | |  |  |  |  |  |  |  |
| ^2^ Wet Weather |  |  |  |  |  |  |  |  |
| ^3^ Greater than 48-hour hold time | |  |  |  |  |  |  |  |

**Table S8. Viral Indicator Concentrations for Facility C**

| **Facility C** | **Male specific (PFU/100 mL)** | | | | **Somatic (PFU/100 mL)** | | | |
| --- | --- | --- | --- | --- | --- | --- | --- | --- |
| **Date** | **Raw Influent** | **Membrane Permeate** | **GAC Effluent** | **Final Effluent** | **Raw Influent** | **Membrane Permeate** | **GAC Effluent** | **Final Effluent** |
| Month 1 (May) | 1.1E+05 | 9.0E+00^1^ | 9.0E+00^1^ | 1.0E+00^1^ | 3.7E+05 | 9.0E+02^1^ | 9.0E+00^1^ | 1.0E+00^1^ |
| Month 2 (June) | 1.4E+05 | 1.0E+00^1^ | 1.0E+00^1^ | 1.0E+00^1^ | 2.2E+05 | 2.0E+00 | 2.0E+00 | 1.0E+00^1^ |
| Month 3 (July) | 1.9E+05 | 1.0E+00^1^ | 1.0E+00^1^ | 1.0E+00^1^ | 2.5E+05 | 2.0E+00 | 1.0E+00^1^ | 1.0E+00^1^ |
| Month 4 (August) | 1.2E+05 | 1.0E+00^1^ | 1.0E+00^1^ | 1.0E+00^1^ | 2.9E+05 | 2.0E+00 | 1.0E+00^1^ | 1.0E+00^1^ |
| Month 5 (September) | 1.2E+05 | 1.0E+00^1^ | 1.0E+00^1^ | 1.0E+00^1^ | 4.5E+05 | 2.0E+00^1^ | 1.8E+01 | 1.0E+00^1^ |
| Month 6 (October)^2^ | 2.7E+04 | 1.0E+00^1^ | 1.0E+00^1^ | 1.0E+00^1^ | 8.6E+04 | 2.0E+00^1^ | 1.0E+00^1^ | 1.0E+00^1^ |
| Month 7 (November) | 3.7E+04 | 1.0E+00^1^ | 1.0E+00^1^ | 1.0E+00^1^ | 1.8E+05 | 2.0E+00 | 1.0E+00^1^ | 1.0E+00^1^ |
| Month 8 (December) | 1.2E+05 | 1.0E+00^1^ | 1.0E+00^1^ | 1.0E+00^1^ | 9.0E+04 | 2.0E+00^1^ | 1.0E+00^1^ | 1.0E+00^1^ |
| Month 9 (January)^3^ | 3.8E+04 | 1.0E+00^1^ | 1.0E+00^1^ | 1.0E+00^1^ | 4.3E+05 | 2.0E+00^1^ | 1.4E+00^1^ | 1.0E+00^1^ |
| Month 10 (February) | 9.4E+04 | 1.0E+00^1^ | 1.0E+00^1^ | 1.0E+00^1^ | 2.3E+05 | 2.0E+00^1^ | 1.0E+00^1^ | 1.0E+00^1^ |
| Month 11 (March) | 6.8E+04 | 1.0E+00^1^ | 1.0E+00^1^ | 1.0E+00^1^ | 1.9E+05 | 2.0E+00^1^ | 1.0E+00^1^ | 1.0E+00^1^ |
| Month 12 (April) | 1.2E+05 | 1.0E+00^1^ | 1.0E+00^1^ | 1.0E+00^1^ | 3.4E+05 | 2.0E+00^1^ | 1.0E+00^1^ | 1.0E+00^1^ |
|  |  |  |  |  |  |  |  |  |
| ^1^Below limit of detection  ^2^Wet Weather  ^3^ Greater than 48-hour hold time | |  |  |  |  |  |  |  |

**Table S9. Bacterial Indicator Concentrations for Facility D**

| **Facility D** | ***E. coli* (CFU/100 mL)** | | | **Enterococci (CFU/100 mL)** | | |
| --- | --- | --- | --- | --- | --- | --- |
| **Date** | **Raw** | **Secondary** | **Disinfected** | **Raw** | **Secondary** | **Disinfected** |
| Month 1 (May) | 8.5E+06 | 2.5E+05 | 1.3E+02 | 1.4E+06 | 6.1E+04 | 1.0E+00^1^ |
| Month 2 (June) | 1.2E+07 | 6.8E+05 | 1.1E+02 | 2.3E+06 | 1.4E+05 | 1.3E+00 |
| Month 3 (July) | 5.2E+06 | 7.0E+05 | 1.7E+02 | 2.5E+06 | 1.3E+05 | 1.0E+01 |
| Month 4 (August) | 1.0E+07 | 1.7E+06 | 1.5E+02 | 1.7E+06 | 4.5E+05 | 2.7E+01 |
| Month 5 (September) | 1.1E+07 | 9.3E+05 | 3.3E+01 | 1.8E+06 | 1.2E+05 | 5.5E+00 |
| Month 6 (October) | 7.1E+06 | 6.0E+05 | 3.1E+01 | 2.1E+06 | 6.8E+04 | 1.0E+01 |
| Month 7 (November) | 6.2E+06 | 7.1E+05 | 1.3E+02 | 3.0E+06 | 1.2E+05 | 9.1E-01 |
| Month 8 (December)^2^ | 2.0E+06 | 2.4E+05 | 5.9E+01 | 4.5E+05 | 9.2E+04 | 1.0E+00^1^ |
| Month 9 (January)^2^ | 5.2E+06 | 2.6E+04 | 1.2E+02 | 8.1E+05 | 8.2E+03 | 9.0E+00 |
| Month 10 (February) | 3.4E+06 | 3.2E+05 | 1.9E+02 | 1.6E+06 | 7.1E+04 | 3.8E+01 |
| Month 11 (March) | 3.1E+06 | 6.8E+04 | 1.8E+01 | 1.4E+06 | 2.7E+04 | 9.1E-01 |
| Month 12 (April)^2^ | 4.9E+06 | 1.5E+05 | 2.5E+01 | 1.4E+06 | 5.2E+04 | 6.3E+00 |
| ^1^Below limit of detection  ^2^Wet Weather |  |  |  |  |  |  |

**Table S10. Viral Indicator Concentrations for Facility D**

| **Facility D** | **Male specific (PFU/100 mL)** | | | **Somatic (PFU/100 mL)** | | |
| --- | --- | --- | --- | --- | --- | --- |
| **Date** | **Raw** | **Secondary** | **Disinfected** | **Raw** | **Secondary** | **Disinfected** |
| Month 1 (May) | 6.1E+04 | 5.6E+03 | 2.5E+01 | 2.8E+05 | 7.9E+04 | 1.4E+01 |
| Month 2 (June) | 1.1E+05 | 2.6E+03 | 1.8E+03 | 1.5E+05 | 1.5E+04 | 5.0E+02 |
| Month 3 (July) | 5.3E+04 | 2.8E+03 | 1.2E+03 | 1.6E+05 | 1.9E+04 | 4.3E+02 |
| Month 4 (August) | 7.6E+04 | 6.0E+03 | 6.4E+03 | 1.0E+05 | 2.5E+04 | 1.1E+03 |
| Month 5 (September) | 8.6E+04 | 2.3E+03 | 2.6E+03 | 2.5E+05 | 4.5E+03 | 6.7E+02 |
| Month 6 (October) | 9.1E+04 | 4.1E+03 | 1.1E+03 | 4.9E+05 | 4.0E+04 | 1.8E+02 |
| Month 7 (November) | 8.5E+04 | 6.5E+03 | 2.4E+03 | 3.6E+05 | 2.9E+04 | 7.0E+02 |
| Month 8 (December)^1^ | 3.5E+04 | 3.4E+03 | 1.3E+03 | 7.3E+04 | 8.2E+03 | 5.8E+02 |
| Month 9 (January)^1^ | 9.7E+04 | 4.3E+03 | 3.8E+03 | 1.8E+05 | 1.5E+04 | 1.3E+03 |
| Month 10 (February) | 7.7E+06 | 4.5E+03 | 5.1E+03 | 1.4E+05 | 1.3E+04 | 1.7E+03 |
| Month 11 (March) | 7.3E+06 | 1.3E+03 | 1.0E+03 | 1.5E+05 | 7.3E+03 | 7.2E+02 |
| Month 12 (April)^1^ | 1.1E+05 | 6.3E+03 | 2.6E+03 | 1.5E+05 | 2.3E+04 | 7.8E+02 |
| ^1^ Wet Weather |  |  |  |  |  |  |

**Table S11. Bacterial Indicator Concentrations for Facility E**

| **Facility E** | ***E. coli* (CFU/100 mL)** | | | | **Enterococci (CFU/100 mL)** | | | |
| --- | --- | --- | --- | --- | --- | --- | --- | --- |
| **Date** | **Raw** | **Primary** | **Secondary** | **Pre-dechlorination** | **Raw** | **Primary** | **Secondary** | **Pre-dechlorination** |
| Month 1 (May) | 6.8E+06 | 6.0E+06 | 6.0E+05 | 3.0E+00 | 2.2E+06 | 8.2E+05 | 1.5E+05 | 1.0E+00^1^ |
| Month 2 (June) | 8.3E+06 | 4.4E+06 | 1.1E+06 | 9.2E+01 | 2.5E+06 | 1.2E+06 | 1.7E+05 | 9.1E-01 |
| Month 3 (July)^2^ | 1.4E+07 | 1.0E+07 | 3.2E+06 | 1.6E+02 | 2.4E+06 | 5.5E+05 | 2.9E+05 | 1.8E+00 |
| Month 4 (August) | 1.2E+07 | 5.9E+06 | 6.1E+05 | 7.4E+01 | 3.5E+06 | 7.9E+05 | 1.2E+05 | 1.8E+00 |
| Month 5 (September) | 1.9E+07 | 1.7E+07 | 8.8E+05 | 1.7E+01 | 2.9E+06 | 6.8E+05 | 1.1E+05 | 9.0E-01^1^ |
| Month 6 (October) | 1.1E+07 | 1.7E+07 | 7.7E+04 | 2.2E+01 | 2.6E+06 | 6.3E+04 | 2.9E+04 | 9.0E-01^1^ |
| Month 7 (November) | 9.9E+06 | 2.0E+07 | 7.4E+05 | 3.6E+01 | 1.2E+06 | 1.0E+06 | 1.2E+05 | 1.0E+00 |
| Month 8 (December) | 4.3E+06 | 5.5E+06 | 6.7E+05 | 6.4E+01 | 6.7E+05 | 4.8E+05 | 1.8E+05 | 3.0E+00 |
| Month 9 (January)^3^ | 9.0E+05 | 1.8E+04 | 1.3E+05 | 5.8E+01 | 4.3E+05 | 2.7E+04 | 5.3E+04 | 2.5E+01 |
| Month 10 (February)^3^ | 5.9E+06 | 6.3E+06 | 1.1E+05 | 1.5E+01 | 1.7E+06 | 1.2E+06 | 7.9E+04 | 2.7E+00 |
| Month 11 (March) | 5.4E+06 | 8.7E+06 | 4.5E+04 | 3.6E+00 | 2.4E+06 | 2.4E+06 | 2.7E+04 | 9.0E-01^1^ |
| Month 12 (April) | 4.4E+06 | 7.6E+06 | 2.3E+05 | 1.9E+01 | 1.6E+06 | 8.5E+05 | 8.5E+04 | 1.3E+00 |
| ^1^ Below limit of detection  ^2^Warm Sample |  |  |  |  |  |  |  |  |
| ^3^ Wet Weather |  |  |  |  |  |  |  |  |

**Table S12. Viral Indicator Concentrations for Facility E**

| **Facility E** | **Male specific (PFU/100 mL)** | | | | **Somatic (PFU/100 mL)** | | | |
| --- | --- | --- | --- | --- | --- | --- | --- | --- |
| **Date** | **Raw** | **Primary** | **Secondary** | **Pre-dechlorination** | **Raw** | **Primary** | **Secondary** | **Pre-dechlorination** |
| Month 1 (May) | 7.3E+04 | 1.2E+05 | 5.0E+03 | 4.9E+03 | 4.1E+05 | 9.1E+04 | 5.5E+04 | 6.1E+03 |
| Month 2 (June) | 1.0E+05 | 8.6E+04 | 1.7E+03 | 2.1E+03 | 5.7E+05 | 2.0E+05 | 7.8E+04 | 2.1E+03 |
| Month 3 (July)^1^ | 1.5E+05 | 8.0E+04 | 1.2E+04 | 1.4E+04 | 3.6E+05 | 8.9E+04 | 3.8E+04 | 8.1E+03 |
| Month 4 (August) | 1.2E+05 | 4.1E+04 | 1.9E+03 | 1.1E+03 | 4.3E+05 | 2.6E+05 | 3.4E+04 | 5.4E+03 |
| Month 5 (September) | 2.4E+05 | 8.3E+04 | 1.6E+03 | 2.0E+03 | 9.5E+05 | 5.2E+05 | 6.9E+04 | 2.2E+04 |
| Month 6 (October) | 8.2E+04 | 1.9E+04 | 1.4E+03 | 7.2E+02 | 4.3E+05 | 1.0E+05 | 1.5E+04 | 5.6E+02 |
| Month 7 (November) | 1.1E+05 | 1.0E+05 | 7.0E+03 | 3.7E+03 | 2.4E+05 | 4.3E+05 | 5.6E+04 | 2.6E+03 |
| Month 8 (December) | 5.9E+04 | 7.1E+04 | 1.5E+03 | 1.4E+03 | 1.3E+05 | 1.1E+05 | 4.1E+04 | 3.1E+03 |
| Month 9 (January)^2^ | 1.9E+04 | 2.4E+04 | 8.7E+02 | 1.0E+03 | 3.0E+04 | 6.3E+03 | 4.9E+03 | 9.1E+02 |
| Month 10 (February)^2^ | 8.9E+04 | 7.6E+04 | 3.2E+03 | 1.6E+03 | 1.9E+05 | 1.8E+05 | 2.8E+04 | 2.5E+03 |
| Month 11 (March) | 9.9E+04 | 8.0E+04 | 1.2E+03 | 1.1E+03 | 5.7E+05 | 1.5E+05 | 3.5E+04 | 2.0E+03 |
| Month 12 (April) | 1.1E+05 | 8.3E+04 | 1.4E+03 | 8.2E+02 | 2.3E+05 | 2.1E+05 | 2.1E+04 | 9.8E+02 |
| ^1^ Warm Sample |  |  |  |  |  |  |  |  |
| ^2^ Wet Weather |  |  |  |  |  |  |  |  |

**Table S13. Bacterial Indicator Concentrations for Facility F**

| **Facility F** | ***E. coli* (CFU/100 mL)** | | | **Enterococci (CFU/100 mL)** | | |
| --- | --- | --- | --- | --- | --- | --- |
| **Date** | **Raw Influent** | **Pre-disinfection Effluent** | **Disinfected Effluent** | **Raw Influent** | **Pre-disinfection Effluent** | **Disinfected Effluent** |
| Month 1 (May) | 4.1E+06 | 9.0E+03 | 1.3E+01 | 1.2E+06 | 2.7E+03 | 1.6E+01 |
| Month 2 (June) | 2.9E+06 | 2.8E+03 | 5.5E+01 | 1.7E+06 | 2.1E+02 | 4.8E+01 |
| Month 3 (July) | 9.0E+06 | 2.0E+04 | 1.3E+02 | 2.0E+06 | 1.8E+03 | 9.1E-01 |
| Month 4 (August)^1^ | 3.3E+06 | 3.0E+04 | 9.1E+01 | 5.6E+05 | 1.3E+04 | 1.6E+02 |
| Month 5 (September)^1^ | 6.1E+06 | 2.3E+04 | 7.4E+01 | 6.3E+05 | 5.6E+03 | 1.4E+02 |
| Month 6 (October) | 4.4E+06 | 1.7E+04 | 1.0E+02 | 1.7E+06 | 3.1E+03 | 1.8E+02 |
| Month 7 (November) | 2.3E+06 | 3.6E+03 | 8.4E+02 | 4.4E+05 | 7.9E+02 | 5.2E+03 |
| Month 8 (December)^2^ | 7.7E+05 | 2.2E+03 | 2.3E+01 | 5.3E+05 | 1.2E+03 | 1.5E+02 |
| Month 9 (January)^2^ | 2.7E+05 | 5.0E+03 | 1.1E+02 | 4.5E+05 | 3.5E+03 | 9.2E+02 |
| Month 10 (February) | 2.2E+06 | 8.6E+03 | 6.4E+00 | 4.7E+05 | 3.1E+03 | 9.1E+00 |
| Month 11 (March)^1,2^ | 5.7E+05 | 2.9E+03 | 5.0E+01 | 3.2E+05 | 1.8E+03 | 6.5E+02 |
| Month 12 (April) | 1.3E+06 | 2.7E+02 | 1.4E+01 | 3.3E+05 | 2.7E+02 | 1.3E+01 |
| ^1^ Time received and temp not recorded | |  |  |  |  |  |
| ^2^ Wet Weather |  |  |  |  |  |  |

**Table S14. Viral Indicator Concentrations for Facility F**

| **Facility F** | **Male specific (PFU/100 mL)** | | | **Somatic (PFU/100 mL)** | | |
| --- | --- | --- | --- | --- | --- | --- |
| **Date** | **Raw Influent** | **Pre-disinfection Effluent** | **Disinfected Effluent** | **Raw Influent** | **Pre-disinfection Effluent** | **Disinfected Effluent** |
| Month 1 (May) | 2.2E+05 | 9.4E+02 | 5.9E+02 | 1.2E+06 | 8.6E+03 | 3.1E+02 |
| Month 2 (June) | 1.7E+05 | 1.0E+03 | 6.2E+02 | 4.7E+05 | 7.2E+03 | 2.3E+02 |
| Month 3 (July) | 4.2E+05 | 3.8E+02 | 2.5E+02 | 5.6E+05 | 1.7E+03 | 1.0E+02 |
| Month 4 (August)^1^ | 1.3E+05 | 1.1E+03 | 6.3E+02 | 3.9E+06 | 1.1E+04 | 2.9E+02 |
| Month 5 (September)^1^ | 2.2E+05 | 1.2E+03 | 1.2E+03 | 7.2E+05 | 4.9E+03 | 5.0E+02 |
| Month 6 (October) | 2.3E+05 | 1.4E+03 | 2.4E+03 | 7.2E+05 | 2.8E+04 | 1.2E+03 |
| Month 7 (November) | 2.8E+05 | 1.2E+04 | 1.4E+04 | 3.5E+05 | 5.8E+04 | 1.4E+04 |
| Month 8 (December)^2^ | 3.8E+04 | 1.9E+03 | 4.9E+02 | 1.9E+05 | 4.4E+03 | 1.4E+03 |
| Month 9 (January)^2^ | 4.5E+04 | 1.0E+03 | 5.0E+02 | 3.8E+05 | 4.0E+03 | 1.1E+03 |
| Month 10 (February) | 7.9E+04 | 6.4E+02 | 2.0E+02 | 8.3E+04 | 2.5E+03 | 1.1E+02 |
| Month 11 (March)^1,2^ | 1.8E+04 | 5.8E+02 | 3.3E+02 | 3.6E+04 | 9.1E+02 | 9.7E+02 |
| Month 12 (April) | 6.9E+04 | 4.9E+02 | 3.7E+02 | 7.6E+04 | 2.0E+03 | 2.4E+02 |
| ^1^ Time received and temp not recorded | |  |  |  |  |  |
| ^2^ Wet Weather |  |  |  |  |  |  |

**Table S15. Bacterial Indicator Concentrations for Facility G**

| **Facility G** | ***E. coli* (CFU/100 mL)** | | | | **Enterococci (CFU/100 mL)** | | | |
| --- | --- | --- | --- | --- | --- | --- | --- | --- |
| **Date** | **Raw Influent** | **Primary Effluent** | **Secondary Effluent** | **Final Effluent** | **Raw Influent** | **Primary Effluent** | **Secondary Effluent** | **Final Effluent** |
| Month 1 (July) | 3.6E+06 | 1.5E+06 | 7.1E+03 | 5.0E+00 | 3.1E+05 | 3.3E+04 | 6.0E+02 | 1.0E+00^1^ |
| Month 2 (August) | 3.8E+06 | 1.7E+06 | 4.3E+03 | 3.0E+00 | 3.3E+05 | 4.3E+04 | 1.7E+02 | 1.0E+00^1^ |
| Month 3 (September) | 2.5E+06 | 1.7E+06 | 4.9E+03 | 2.0E+00 | 3.7E+05 | 6.9E+04 | 1.0E+02^1^ | 1.0E+00^1^ |
| Month 4 (October) | 3.7E+06 | 2.3E+06 | 6.5E+03 | 3.0E+00 | 3.4E+05 | 4.8E+04 | 1.0E+02 | 1.0E+00 |
| Month 5 (November) | 3.1E+06 | 1.4E+06 | 5.1E+03 | 2.0E+00 | 3.3E+05 | 4.0E+04 | 1.0E+02 | 1.0E+00^1^ |
| Month 6 (December) | 2.9E+06 | 8.0E+05 | 1.8E+04 | 7.0E+00 | 4.0E+05 | 3.6E+04 | 2.0E+02 | 3.0E+00 |
| Month 7 (January) | 1.7E+06 | 1.8E+06 | 8.2E+03 | 2.0E+00 | 4.8E+05 | 7.6E+04 | 5.0E+02 | 1.0E+00^1^ |
| Month 8 (February) | 3.0E+06 | 2.9E+06 | N/A | N/A | 3.1E+05 | 2.1E+05 | N/A | N/A |
| Month 9 (March) | 3.8E+06 | 3.1E+06 | 1.9E+04 | 1.0E+00 | 5.8E+05 | 6.1E+04 | 6.0E+02 | 1.0E+00^1^ |
| Month 10 (April) | 2.1E+06 | 1.8E+06 | 2.3E+04 | 2.0E+00 | 6.0E+05 | 7.0E+04 | 8.0E+02 | 1.0E+00^1^ |
| Month 11 (May) | 3.4E+06 | 2.6E+06 | 2.4E+04 | 1.0E+00 | 4.0E+05 | 6.6E+04 | 6.0E+02 | 1.0E+00^1^ |
| Month 12 (June) | 3.0E+06 | 1.9E+06 | 2.7E+04 | 2.0E+00 | 3.1E+05 | 6.1E+04 | 1.1E+03 | 1.0E+00^1^ |

^1^ Below limit of detection

**Table S16. Viral Indicator Concentrations for Facility G**

| **Facility G** | **Male specific (PFU/100 mL)** | | | | **Somatic (PFU/100 mL)** | | | |
| --- | --- | --- | --- | --- | --- | --- | --- | --- |
| **Date** | **Raw Influent** | **Primary Effluent** | **Secondary Effluent** | **Final Effluent** | **Raw Influent** | **Primary Effluent** | **Secondary Effluent** | **Final Effluent** |
| Month 1 (July) | 1.0E+05 | 2.3E+03 | 1.4E+01 | 1.0E+00^1^ | 9.5E+03 | 6.4E+03 | 5.5E+01 | 2.0E+00 |
| Month 2 (August) | 3.1E+05 | 2.6E+04 | 1.8E+01 | 1.0E+01 | 7.1E+04 | 4.7E+04 | 6.1E+02 | 3.1E+01 |
| Month 3 (September) | 2.3E+05 | 3.2E+04 | 3.5E+01 | 2.0E+00 | 2.8E+05 | 3.8E+04 | 2.1E+02 | 4.0E+00 |
| Month 4 (October) | 5.5E+04 | 5.0E+03 | 7.3E+01 | 3.3E+01 | 7.7E+04 | 1.8E+04 | 9.5E+02 | 3.9E+01 |
| Month 5 (November) | 9.8E+04 | 8.6E+03 | 3.5E+01 | 9.0E+00 | 4.8E+04 | 1.1E+04 | 6.1E+02 | 1.8E+01 |
| Month 6 (December) | 7.4E+04 | 1.8E+04 | 9.5E+01 | 2.5E+01 | 5.7E+04 | 1.8E+04 | 5.5E+02 | 6.5E+01 |
| Month 7 (January) | 2.1E+05 | 5.6E+04 | 4.6E+02 | 2.2E+02 | 3.1E+04 | 6.4E+03 | 4.6E+02 | 5.7E+01 |
| Month 8 (February) | 1.9E+05 | 6.0E+04 | N/A | N/A | 6.6E+04 | 2.0E+04 | N/A | N/A |
| Month 9 (March) | 1.3E+05 | 2.5E+04 | 4.3E+02 | 3.5E+02 | 1.2E+05 | 2.4E+04 | 6.6E+05 | 2.9E+02 |
| Month 10 (April) | 1.2E+05 | 2.1E+04 | 1.5E+02 | 1.4E+02 | 6.8E+04 | 1.5E+04 | 8.4E+02 | 1.7E+02 |
| Month 11 (May) | 1.3E+05 | 7.0E+03 | 2.2E+02 | 1.1E+02 | 1.0E+05 | 3.2E+04 | 1.2E+03 | 1.7E+02 |
| Month 12 (June) | 1.1E+05 | 5.6E+04 | 3.3E+02 | 1.7E+02 | 5.0E+05 | 1.6E+05 | 1.8E+03 | 1.0E+02 |

^1^ Below limit of detection

**Table S17. Bacterial Indicator Concentrations for Facility H**

| **Facility H** | ***E. coli* (CFU/100 mL)** | | | | **Enterococci (CFU/100 mL)** | | | |
| --- | --- | --- | --- | --- | --- | --- | --- | --- |
| **Date** | **Raw Influent** | **Primary Effluent** | **Secondary Effluent** | **Final Effluent** | **Raw Influent** | **Primary Effluent** | **Secondary Effluent** | **Final Effluent** |
| Month 1 (July) | 6.1E+06 | 2.0E+06^2^ | 3.3E+05 | 4.2E+01 | 2.3E+06 | 2.0E+06^2^ | 1.4E+04 | 1.1E+01 |
| Month 2 (August) | 6.3E+06 | 2.3E+06 | 1.2E+05 | 2.0E+00 | 1.4E+06 | 1.0E+05 | 1.2E+04 | 1.0E+00^1^ |
| Month 3 (September) | 3.7E+06 | 1.9E+06 | 7.0E+02 | 5.0E+00 | 6.2E+05 | 1.3E+05 | 1.0E+03^1^ | 1.0E+00 |
| Month 4 (October) | 2.9E+06 | 2.4E+06 | 4.2E+04 | 5.8E+01 | 2.6E+05 | 1.9E+04 | 1.7E+03 | 7.0E+00 |
| Month 5 (November) | 1.4E+06 | 1.3E+06 | 1.5E+04 | 4.0E+00 | 2.9E+05 | 4.9E+04 | 1.6E+03 | 1.0E+00 |
| Month 6 (December) | 1.3E+06 | 1.5E+06 | 3.0E+03 | 1.0E+00 | 2.5E+05 | 4.2E+04 | 1.0E+03^1^ | 1.0E+00^1^ |
| Month 7 (January) | 1.0E+04^1^ | 7.0E+05 | 2.7E+04 | 1.0E+01 | 1.4E+05 | 5.6E+04 | 7.2E+03 | 1.0E+00^1^ |
| Month 8 (February) | 1.2E+06 | 9.4E+05 | 5.0E+03 | 1.0E+00 | 2.0E+05 | 1.0E+05 | 1.1E+03 | 1.0E+00 |
| Month 9 (March) | 1.3E+06 | 1.4E+06 | 1.0E+03 | 3.0E+00 | 5.2E+05 | 1.0E+05 | 1.0E+03^1^ | 1.0E+01 |
| Month 10 (April) | 2.6E+06 | 1.9E+06 | 3.1E+04 | 1.0E+00 | 4.2E+05 | 1.6E+05 | 6.3E+03 | 1.0E+00^1^ |
| Month 11 (May) | 3.4E+06 | 2.4E+06 | 2.2E+04 | 4.0E+00 | 6.8E+04 | 6.0E+04 | 2.6E+03 | 1.0E+00^1^ |
| Month 12 (June) | 3.8E+06 | 2.6E+06 | 1.6E+04 | 2.0E+00 | 5.4E+05 | 6.7E+04 | 5.4E+03 | 1.0E+00^1^ |

^1^ Below limit of detection
^2^ Above upper limit of quantification

**Table 18. Viral Indicator Concentrations for Facility H**

| **Facility H** | **Male specific (PFU/100 mL)** | | | | **Somatic (PFU/100 mL)** | | | |
| --- | --- | --- | --- | --- | --- | --- | --- | --- |
| **Date** | **Raw Influent** | **Primary Effluent** | **Secondary Effluent** | **Final Effluent** | **Raw Influent** | **Primary Effluent** | **Secondary Effluent** | **Final Effluent** |
| Month 1 (July) | 7.5E+04 | 4.1E+03 | 8.6E+01 | 1.6E+01 | 2.7E+04 | 8.6E+03 | 5.0E+02 | 4.0E+00 |
| Month 2 (August) | 6.1E+04 | 1.4E+03 | 1.8E+01 | 5.0E+00 | 3.8E+05 | 1.0E+05 | 1.2E+03 | 7.0E+00 |
| Month 3 (September) | 8.5E+04 | 2.4E+04 | 4.1E+01 | 3.0E+00 | 8.8E+04 | 3.0E+04 | 7.7E+01 | 8.0E+00 |
| Month 4 (October) | 5.2E+04 | 8.6E+03 | 2.3E+01 | 8.0E+00 | 6.3E+04 | 2.4E+04 | 8.5E+02 | 5.9E+01 |
| Month 5 (November) | 9.2E+04 | 3.0E+04 | 9.5E+01 | 2.7E+01 | 3.8E+04 | 3.1E+04 | 1.8E+03 | 4.1E+01 |
| Month 6 (December) | 5.7E+04 | 2.5E+04 | 4.5E+01 | 3.0E+00 | 3.2E+04 | 1.4E+04 | 9.2E+02 | 7.0E+00 |
| Month 7 (January) | 4.0E+04 | 8.6E+04 | 1.9E+02 | 7.8E+01 | 9.5E+03 | 3.6E+04 | 5.2E+02 | 7.8E+01 |
| Month 8 (February) | 7.5E+04 | 4.4E+04 | 1.7E+02 | 3.3E+01 | 4.5E+04 | 3.3E+04 | 1.3E+03 | 2.3E+01 |
| Month 9 (March) | 1.2E+05 | 4.9E+04 | 6.8E+01 | 1.0E+01 | 7.5E+04 | 5.1E+04 | 8.9E+02 | 6.4E+01 |
| Month 10 (April) | 8.7E+04 | 5.6E+04 | 1.3E+03 | 7.2E+01 | 4.0E+04 | 3.8E+04 | 1.0E+03 | 4.3E+01 |
| Month 11 (May) | 4.6E+04 | 2.9E+04 | 1.3E+02 | 4.2E+01 | 8.6E+04 | 4.7E+04 | 1.1E+03 | 5.4E+01 |
| Month 12 (June) | 8.1E+04 | 3.3E+04 | 2.2E+02 | 4.5E+01 | 2.7E+05 | 4.3E+04 | 2.1E+03 | 5.7E+01 |

**Table 19. Bacterial Indicator Concentrations for Facility I**

| **Facility I** | ***E. coli* (CFU/100 mL)** | | | | **Enterococci (CFU/100 mL)** | | | |
| --- | --- | --- | --- | --- | --- | --- | --- | --- |
| **Date** | **Raw Influent** | **Primary Effluent** | **Secondary Effluent** | **Final Effluent** | **Raw Influent** | **Primary Effluent** | **Secondary Effluent** | **Final Effluent** |
| Month 1 (July) | 3.6E+06 | 2.3E+06 | 2.0E+05^2^ | 1.0E+00^1^ | 5.2E+05 | 3.7E+05 | 1.1E+04 | 1.0E+00^1^ |
| Month 2 (August) | 1.8E+08 | 2.0E+07 | 1.6E+03 | 2.0E+00 | 1.3E+06 | 1.9E+05 | 2.2E+03 | 2.0E+00 |
| Month 3 (September) | 1.4E+06 | 2.2E+06 | 6.2E+04 | 9.0E+00 | 2.4E+05 | 1.1E+05 | 2.4E+03 | 1.0E+00 |
| Month 4 (October) | 2.0E+06 | 2.2E+06 | 7.1E+04 | 3.0E+00 | 2.3E+05 | 5.0E+04 | 2.9E+03 | 6.0E+00 |
| Month 5 (November) | 1.5E+06 | 1.2E+06 | 2.8E+04 | 5.0E+00 | 2.7E+05 | 1.4E+05 | 1.1E+03 | 1.0E+00^1^ |
| Month 6 (December) | 3.6E+06^1^ | 1.0E+04 | 2.9E+03 | 2.0E+00 | 4.0E+05 | 2.6E+05 | 1.0E+03^1^ | 1.0E+00^1^ |
| Month 7 (January) | 5.9E+05 | 1.1E+06^1^ | 1.0E+03^1^ | 1.0E+00 | 1.2E+05 | 1.6E+05 | 1.0E+03^1^ | 3.0E+00 |
| Month 8 (February) | 8.5E+05 | 8.0E+05 | 3.2E+04 | 1.0E+00 | 2.8E+05 | 2.5E+05 | 5.5E+03 | 1.0E+00^1^ |
| Month 9 (March) | 2.7E+06 | 2.0E+06^1^ | 1.0E+03^1^ | 1.0E+00 | 4.7E+05 | 2.9E+05 | 1.0E+03^1^ | 2.0E+00 |
| Month 10 (April) | 1.5E+06 | 1.5E+06 | 5.5E+02 | 2.0E+00 | 1.8E+05 | 1.1E+05 | 1.0E+02^1^ | 6.0E+00 |
| Month 11 (May) | 1.9E+06 | 2.6E+05 | 2.6E+03 | 5.0E+00 | 3.7E+05 | 1.7E+05 | 1.4E+03 | 1.0E+00^1^ |
| Month 12 (June) | 2.0E+06 | 2.1E+06 | 3.1E+03 | 4.5E+01 | 2.6E+05 | 2.1E+05 | 1.0E+03^1^ | 8.0E+00 |

^1^ Below limit of detection
^2^ Above upper limit of quantification

**Table 20. Viral Indicator Concentrations for Facility I**

| **Facility I** | **Male specific (PFU/100 mL)** | | | | **Somatic (PFU/100 mL)** | | | |
| --- | --- | --- | --- | --- | --- | --- | --- | --- |
| **Date** | **Raw Influent** | **Primary Effluent** | **Secondary Effluent** | **Final Effluent** | **Raw Influent** | **Primary Effluent** | **Secondary Effluent** | **Final Effluent** |
| Month 1 (July) | 6.0E+04 | 1.3E+05 | 2.1E+03 | 1.4E+03 | 5.7E+03 | 7.8E+03 | 1.2E+02 | 1.0E+00^1^ |
| Month 2 (August) | 3.0E+04 | 1.2E+05 | 1.0E+02 | 1.0E+00^1^ | 6.5E+04 | 6.1E+04 | 1.3E+03 | 2.0E+00 |
| Month 3 (September) | 2.8E+04 | 2.6E+04 | 3.1E+02 | 3.4E+01 | 6.1E+04 | 4.2E+04 | 5.6E+02 | 7.7E+01 |
| Month 4 (October) | 1.6E+05 | 3.6E+04 | 8.6E+01 | 6.0E+00 | 3.3E+04 | 5.0E+04 | 1.0E+03 | 1.5E+01 |
| Month 5 (November) | 7.6E+04 | 3.5E+03 | 1.9E+02 | 3.0E+00 | 3.8E+04 | 4.3E+04 | 1.2E+03 | 4.0E+00 |
| Month 6 (December) | 2.6E+04 | 2.5E+04 | 2.3E+01 | 2.0E+00 | 3.3E+04 | 3.6E+04 | 1.9E+02 | 1.4E+01 |
| Month 7 (January) | 1.2E+04 | 3.1E+04 | 4.1E+01 | 1.2E+01 | 1.4E+04 | 2.2E+04 | 2.6E+02 | 1.0E+00 |
| Month 8 (February) | 5.1E+04 | 6.6E+04 | 1.6E+02 | 2.0E+00 | 3.9E+04 | 4.1E+04 | 1.5E+03 | 1.0E+00^1^ |
| Month 9 (March) | 7.5E+04 | 2.6E+04 | 2.5E+02 | 1.0E+00^1^ | 7.1E+04 | 5.8E+04 | 1.8E+02 | 1.0E+00 |
| Month 10 (April) | 6.6E+04 | 5.7E+04 | 4.5E+00^1^ | 1.0E+00^1^ | 7.1E+04 | 3.2E+04 | 4.5E+00^1^ | 1.0E+00^1^ |
| Month 11 (May) | 2.1E+04 | 2.2E+04 | 1.8E+01 | 1.0E+00 | 1.1E+05 | 1.1E+05 | 7.4E+02 | 1.0E+00^1^ |
| Month 12 (June) | 5.5E+04 | 5.1E+04 | 6.8E+01 | 1.0E+00^1^ | 4.6E+04 | 6.2E+04 | 1.1E+03 | 1.0E+02 |

^1^ Below limit of detection

**Table S21.** Indicator Organism Mean and Standard Deviation Primary Treatment Log Reductions^1^

| **Facility** | **Clarifier Type** | ***E. coli* Reduction** | **Enterococci Reduction** | **Somatic Reduction** | **Male specific Reduction** |
| --- | --- | --- | --- | --- | --- |
| Facility A | Conventional | 0.0+0.1 | 0.2+0.3 | 0.3+0.4 | 0.1+0.2 |
| Facility E | Conventional | 0.1+0.5 | 0.5+0.5 | 0.3+0.3 | 0.2+0.2 |
| Facility G | Conventional | 0.2+0.2 | 0.9+0.1 | 0.6+0.2 | 0.9+0.4 |
| Facility H | Conventional | 0.0+0.6 | 0.6+0.4 | 0.3+0.4 | 0.5+0.5 |
| Facility I | Conventional | 0.4+0.8 | 0.3+0.3 | 0.0+0.2 | 0.1+0.5 |

^1^ Primary effluent samples were not collected for WRRFs B and C due to tertiary process sampling, and WRRFs D and F do not have primary treatment.

**Table S22.** Indicator Organism Mean and Standard Deviation Secondary Treatment Log Reductions (Reductions with the Asterisk are for Facilities without Primary Treatment)^2^

| **Facility** | **Secondary Process** | ***E. coli* Reduction** | **Enterococci Reduction** | **Somatic Reduction** | **Male specific Reduction** |
| --- | --- | --- | --- | --- | --- |
| Facility A | 5 stage BNR | 2.4+0.3 | 2.2+0.9 | 1.7+0.6 | 3.1+0.9 |
| Facility D* | Step Aeration AS | 1.2+0.5 | 1.3+0.4 | 1.0+0.3 | 1.5+0.7 |
| Facility E | Pure O_2_ AS | 1.0+0.8 | 0.6+0.4 | 0.6+0.3 | 1.4+0.3 |
| Facility F* | SBR | 2.5+0.4 | 2.6+0.6 | 1.8+0.5 | 2.1+0.5 |
| Facility G | IFAS | 2.2+0.3 | 2.2+0.4 | 1.3+1.0 | 2.2+0.5 |
| Facility H | 3 cell BNR | 2.1+0.8 | 1.5+0.5 | 1.6+0.4 | 2.4+0.4 |
| Facility I | 3 cell BNR (A2O) | 2.2+1.1 | 2.1+0.5 | 2.0+0.6 | 2.6+0.8 |

^2^ Primary effluent samples were not collected for WRRFs B and C due to tertiary process sampling.

**Table S23.** Indicator Organism Mean and Standard Deviation Disinfection Log Reductions (Asterisk Indicates that the Log-Reduction Value Was Limited by the Pre-Disinfection Indicator Organism Concentration)

| **Facility** | **Disinfectant** | **Chlorine Species** | ***E. coli* Reduction** | **Enterococci Reduction** | **Somatic Reduction** | **Male specific Reduction** |
| --- | --- | --- | --- | --- | --- | --- |
| Facility A* | LP-UV | #N/A | 3.0+0.6 | 2.4+2.7 | 3.1+0.8 | 1.5+1.0 |
| Facility B* | Ozone | #N/A | 2.6+0.6 | 2.4+0.8 | 2.4+0.4 | 1.4+0.5 |
| Facility C* | MP-UV | #N/A | 0.2+0.6 | 0.2+0.6 | 0.3+0.5 | 0.1+0.3 |
| Facility D | Chlorine | Combined | 3.6+0.6 | 4.3+0.7 | 1.6+0.9 | 0.4+0.7 |
| Facility E | Chlorine | Combined | 4.1+0.6 | 4.8+0.6 | 1.0+0.4 | 0.1+0.2 |
| Facility F | PAA | #N/A | 2.1+0.7 | 1.4+1.2 | 1.1+0.4 | 0.2+0.3 |
| Facility G | Chlorine | Combined | 3.7+0.5 | 2.5+0.4 | 1.4+0.7 | 0.5+0.4 |
| Facility H | Chlorine | Combined | 3.5+0.8 | 3.2+0.6 | 1.5+0.5 | 0.7+0.3 |
| Facility I | Chlorine | Combined/Free | 3.4+1.0 | 2.9+0.7 | 2.0+0.9 | 1.3+0.7 |

**Table S24.** Spearman’s correlation between enteric viruses and fecal indicators. Significant correlations are in bold

| ***Raw Influent*** | 1 | 2 | 3 | 4 | 5 | 6 |
| --- | --- | --- | --- | --- | --- | --- |
| (1) Male-Specific |  |  |  |  |  |  |
| (2) Somatic | 0.18 |  |  |  |  |  |
| (3) *E. coli* | 0.17 | 0.13 |  |  |  |  |
| (4) Enterococci | 0.29 | **0.46** | 0.27 |  |  |  |
| (5) Adenovirus | 0.01 | -0.09 | -0.17 | -0.17 |  |  |
| (6) Norovirus GI | 0.07 | 0.05 | **-0.34** | 0 | 0.29 |  |
| (7) Norovirus GII | -0.01 | -0.2 | **-0.32** | **-0.17** | **0.52** | **0.64** |
| ***Final Effluent*** |  |  |  |  |  |  |
| (1) Male-Specific |  |  |  |  |  |  |
| (2) Somatic | **0.63** |  |  |  |  |  |
| (3) *E. coli* | **-0.31** | 0.07 |  |  |  |  |
| (4) Enterococci | **-0.39** | -0.15 | **0.33** |  |  |  |
| (5) Adenovirus | 0.29 | 0.26 | 0.28 | -0.08 |  |  |
| (6) Norovirus GI | **0.67** | **0.55** | **-0.44** | -0.17 | 0.1 |  |
| (7) Norovirus GII | **0.59** | **0.45** | **-0.35** | -0.09 | 0.28 | **0.82** |
